# The structural impact of DNA mismatches

**DOI:** 10.1093/nar/gkv254

**Published:** 2015-03-27

**Authors:** Giulia Rossetti, Pablo D. Dans, Irene Gomez-Pinto, Ivan Ivani, Carlos Gonzalez, Modesto Orozco

**Affiliations:** 1 Joint BSC-CRG-IRB Program on Computational Biology, Institute for Research in Biomedicine (IRB Barcelona), Baldiri Reixac, 10, Barcelona 08028, Spain; 2 Computational Biophysics, German Research School for Simulation Sciences (Joint venture of RWTH Aachen University and Forschungszentrum Jülich, Germany), D-52425 Jülich, Germany and Institute for Advanced Simulation IAS-5, Computational Biomedicine, Forschungszentrum Jülich, D-52425 Jülich, Germany; 3 Juelich Supercomputing Center (JSC), Forschungszentrum Jülich, Jülich, Germany; 4 Instituto de Química Física Rocasolano, CSIC, C/Serrano 119, Madrid 28006, Spain; 5 Departament de Bioquímica i Biologia Molecular, Facultat de Biologia, Universitat de Barcelona, Avgda Diagonal 647, Barcelona 08028, Spain

## Abstract

The structure and dynamics of all the transversion and transition mismatches in three different DNA environments have been characterized by molecular dynamics simulations and NMR spectroscopy. We found that the presence of mismatches produced significant local structural alterations, especially in the case of purine transversions. Mismatched pairs often show promiscuous hydrogen bonding patterns, which interchange among each other in the nanosecond time scale. This therefore defines flexible base pairs, where breathing is frequent, and where distortions in helical parameters are strong, resulting in significant alterations in groove dimension. Even if the DNA structure is plastic enough to absorb the structural impact of the mismatch, local structural changes can be propagated far from the mismatch site, following the expected through-backbone and a previously unknown through-space mechanism. The structural changes related to the presence of mismatches help to understand the different susceptibility of mismatches to the action of repairing proteins.

## INTRODUCTION

DNA mismatch (MM) is a DNA defect occurring when two non-complementary bases are aligned in the same base-pair step of a duplex DNA (1). MM can appear during replication of DNA (2), heteroduplex formation (3), mutagenic chemicals, ionizing radiation, or spontaneous deamination (4). While MMs are well tolerated in RNA, they are quickly corrected in DNA by the mismatch repair (MMR) proteins. Failures in detecting or correcting the lesion give rise to genetic mutations (4–7). In fact, MMs have been associated with 10–30% of spontaneous cancers in various tissues, as well as in some hereditary cancers such as the colorectal one (8,9).

A MM is defined as ‘transduction’, when formed by non-complementary purine(Pur)·pyrimidine(Pyr) bases, and ‘transversion’ in the case of Pur·Pur or Pyr·Pyr pairs (1). MMs introduce major changes in the canonical (Watson-Crick) recognition rules, and are expected to produce major alterations in the structure and stability of the DNA helix, especially in the proximity of the MM site (10–15). Suggestions have been made on the existence of a weak correlation between such structural and stability changes and the efficiency of MMR proteins to recognize and repair the MM (16–18). This in turn has been related to the different binding affinity of mutated DNAs for MutS protein (19–22) (or its homologous in human, MutSα (19)), which recognizes mispaired nucleotides and allows further action of MMR proteins. However, a complete atlas of the structure and dynamics of the DNA MMs, which hinders the elucidation of how MM-related structural changes are connected with repairing efficiency, is so far lacking. Such knowledge would help in understanding why, for example, Pur·Pur MMs are better repaired than the Pyr·Pyr ones, or why the sequence environment of the lesion influences the ability of the MMR to detect the lesion (12,13,23–29).

We present here the first comprehensive study of the structure of MM-containing DNA duplexes. By using state-of-the-art molecular dynamics (MD) simulations complemented with NMR spectroscopy, we analyzed all the 12 possible MMs in three different sequence environments. We found that there is not a single path of MM-induced changes, since different types of MMs modify DNA properties in different ways. However, all the analyzed MMs displayed an *all-anti* arrangement. Depending on the environment, local structural distortion, induced by the lesion, can propagate to relatively distant regions, acting probably as ‘antenna’ for the MMR proteins. Even though the mechanism for recognition of lesions by MutS (or its homologues) is complex and multifactorial, we found that basic description of the lesion in terms of structure (groove alterations) and dynamics (breathing frequency) can help understanding, at least partially, the relative susceptibility of different MMs to the action of MutS (or its homologues).

## MATERIALS AND METHODS

### METHODS

#### Studied systems

**Figure 1.**
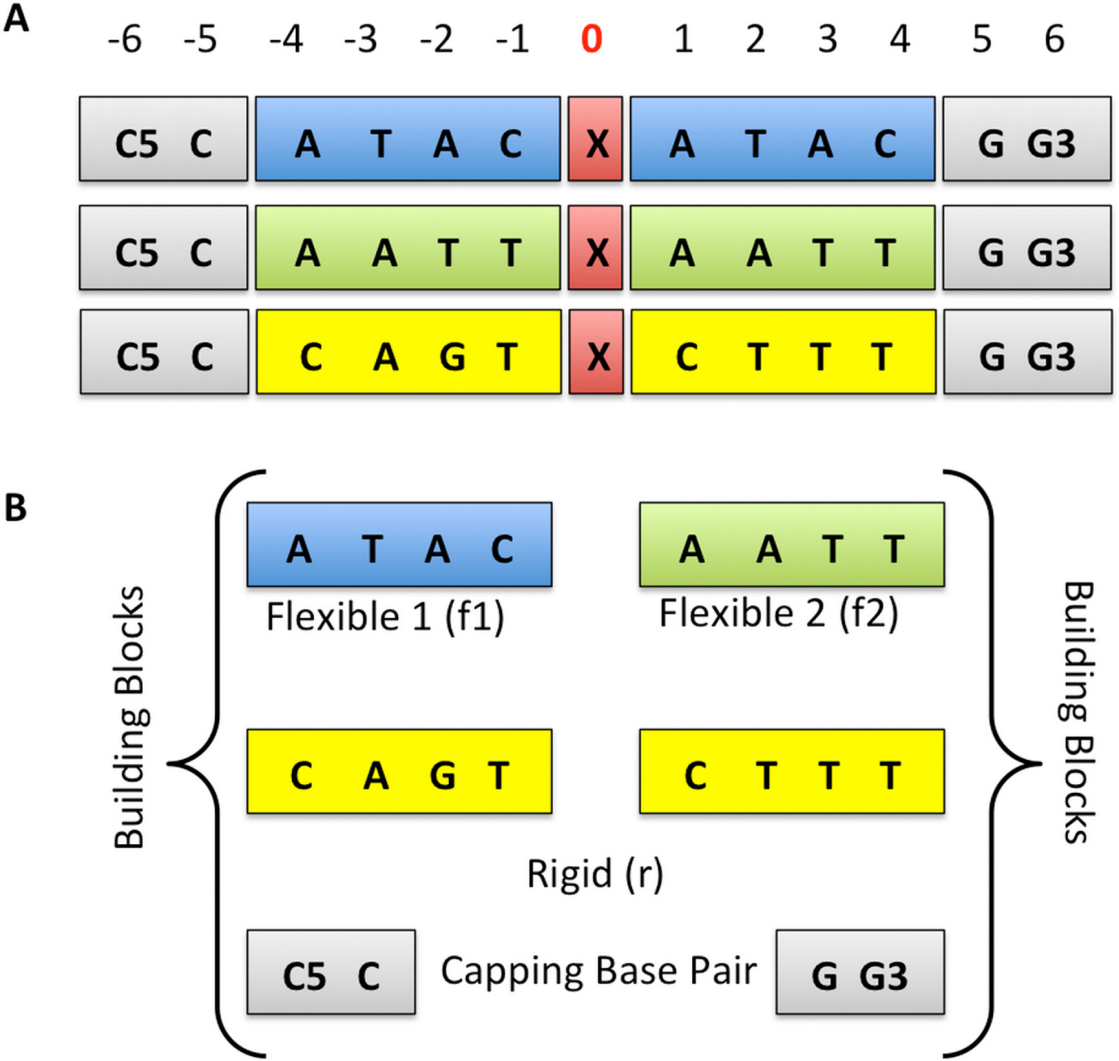
System architecture. (**A**) Types of sequences built. The mutated position is indicated with ‘X’ character and red background. (**B**) Building Blocks Used.

We considered the twelve MMs (A·A, A·C, A·G, C·A, C·C, C·T, G·A, G·G, G·T, T·C, T·G and T·T) placed in the center of three different 13-mer duplexes selected to obtain stable ends (made with C·G pairs) and different flexibility environments: two flexible, and one rigid. The flexible blocks were designed on the basis of DNA elastic properties emerged in previous works: (i) Pyr·Pur base pair steps (bps), as CA and TA, are known to be the most flexible steps out of the 10 unique bps as shown in the Ascona B-DNA Consortium (ABC) publications (30,31), and previous studies on nucleic acids flexibility (32–34). So we *a priori* assume that any mismatch surrounded by Pyr and Pur (in the 5′ → 3′ direction) will be subject to a flexible environment. (ii) At the base pair level, we considered that A-T base pairs have more breathing than C-G, a feature that will clearly add more flexibility to the sequence. Following these two criteria, we further analyzed our local database of duplex B-DNA trajectories looking for sequences showing rigidity or flexibility in terms of global helical bend, opening, and minor groove width, i.e the main distortions expected for MutS (and its homologues) binding (19). We therefore selected the final two flexible blocks: ATAC and AATT (f1 and f2). The rigid sequence is actually the most common (consensus) sequence found in the Protein Data Bank flanking DNA in the presence of a lesion (mismatch, break, gap, abasic site, etc.). To verify that our original selection was correct, we performed four control simulations (200 ns long) for each of the selected duplex (i.e. 3 × 4 trajectories), considering the four canonical pairings d(G·C),d(A·T),d(C·G) and d(T·A) placed at the central position. For each trajectory we computed the stiffness constants associated to global helical bending, opening and minor groove width in the center of the helix (the relevant perturbations in MutS recognition). Results in Supplementary Table S1 illustrate that our original choice was correct and that, d(CCATACXATACGG) and d(CCAATTXAATTGG) are good examples of globally flexible helices (in terms of MutS-relevant distortions), while d(CCCAGTXCTTTGG) is a good example of rigid duplex. These three helices were used as containers for the MMs, performing a total of 48 simulations (12 MMs in three different helical environments, plus the 4 × 3 control simulations of the canonically paired B-DNA, Figure 1).

#### System preparation and production trajectories

Structural models for all the potential systems in a B-like geometry were generated using NucGen (35). All the systems were solvated in a truncated octahedral box of explicit water large enough as to guarantee a minimum distance of at least 11 Å from the DNA to any face of the periodic box. Systems were neutralized by adding Na^+^ cations, optimized, thermalized and equilibrated using our standard protocol (36,37), followed by additional 2 ns post-equilibration trajectory. Equilibrated systems were then subjected to at least 200 ns of unrestrained MD simulation in the NPT ensemble (*P* = 1 atm, *T* = 298 K), using the Nosé-Hoover thermostat (38), and Andersen-Parrinello barostat (39,40). Periodic boundary conditions and Particle Mesh Ewald (real space cutoff 12 Å and grid spacing 1.2 Å) were used to account for remote electrostatic interactions (41). Van der Waals contacts were truncated at the real space cutoff. All bonds containing hydrogen were constrained using LINCs (42), which allowed us to use an integration step of 2 fs. The parmbsc0 modification (43) of parm99 force-field (44) was used to describe DNA. Water was described using TIP3P (45) model, while Dang's parameters were used to describe ions (46). MD trajectories were obtained using Gromacs v.4.5.5 program (47,48). To test the good sampling of our simulations and the statistical relevance of the calculated properties, we extend the simulations of GGf2 and AAf2 up to 700 ns.

#### Free energy surface calculations

As unbiased MD simulations failed to sample spontaneous *syn/anti* transitions of the glycosidic torsion angle (χ) at the MM position (see below), we forced the transition in the G·G MM (where χ transition is more likely to occur) computing the associated free energy. For this purpose we first performed adiabatic bias molecular dynamic (ABMD) (49), to obtain a reasonable pathway of the transition (Supplementary Figures S1 and S2 in Supporting Information, SI hereafter), which were then used to set up metadynamics calculations (50). ABMD allows a system to evolve toward a target value by one or few collective variables (CVs) by using a harmonic potential moving with the thermal fluctuations of the CVs (49). Since we were interested in the *anti*-to-*syn* transition, the χ angle of the purine, which defines the *cis* and *trans* configuration, was used as CV (51). The simulation was carried out using PLUMED 1.3 plugin (52) in combination with GROMACS 4.5.5 (47,48), employing the same computational setup described above for the production trajectories. The transition was detected in a very short ABMD simulation period (around 200 ps, see Supplementary Figure S1 in SI). However more than 50 ns were needed for the stabilization of the *syn* hydrogen bond (HB) pattern (see Supplementary Figure S2 in SI), which suggested that using two rather than one CV to guide the transition would be a safer strategy. Therefore a metadynamics (50) simulation was performed as a function of two CVs, the χ angle and the G(H1)-G’(O6) HB of *syn* conformation, which should be relevant for describing the dissociation process. The Well-Tempered (WT) method (53) was used to accelerate convergence. The widths of Gaussians were chosen to be ∼1/3 of the typical fluctuations of the CVs during the MD simulation (54). The height of the Gaussian functions was set to ∼ 0.024 kcal/mol, with a deposition time equal to 1 ps. The same force field as for the unbiased simulations was employed. The simulation requested around 500 ns of sampling. The error on the calculated free-energy profile was estimated as in Branduardi *et al*. (55). The metadynamics free energy profile was finally corrected for potential errors in the representation of glycosidic torsion of parmbsc0 (56) by adding high level corrections derived from MP2(aug-cc-pVDZ)/CBS-CCSD(T) calculations.

#### Trajectory analyses

The methodological details of structural and energetic analysis here performed are reported in detail in SI.

#### NMR analysis

NMR spectroscopy studies were performed to confirm the prevalence of *anti* conformations of purines in MMs and to evaluate the extension of the structural distortion induced by the lesion. Samples of the duplexes were suspended in 500 μl of either D_2_O or H_2_O/D_2_O 9:1 in 100 mM NaCl and 25 mM sodium phosphate, pH 7. NMR spectra were acquired in Bruker Advance spectrometers operating at 600 or 800 MHz, and processed with Topspin software (57). DQF-COSY, TOCSY and NOESY experiments were recorded in D_2_O. The NOESY spectra were acquired with mixing times of 150 and 250 ms, and the TOCSY spectra were recorded with standard MLEV17 spinlock sequence, and 80 ms mixing time. NOESY spectra in H_2_O were acquired with 150 ms mixing times. In 2D experiments in H_2_O, water suppression was achieved by including a WATERGATE (58) module in the pulse sequence prior to acquisition. Two-dimensional experiments in D_2_O were carried out at 25ºC, whereas spectra in H_2_O were recorded at 5ºC to reduce the exchange with water. Spectral assignment was carried out following standard methods with program Sparky (59). Quantitative distance constraints were obtained from NOESY experiments by using a complete relaxation matrix analysis with the program MARDIGRAS (60). Error bounds in the interprotonic distances were estimated by carrying out several MARDIGRAS calculations with different initial models, mixing times and correlation times. Standard A- and B-form duplexes were used as initial models, and three correlation times (3.0, 5.0 and 7.0 ns) were employed, assuming an isotropic motion for the molecule. Experimental intensities were recorded at different mixing times (150 and 250 ms). Final constraints were obtained by averaging the upper and lower distance bounds in all the MARDIGRAS runs.

## RESULTS AND DISCUSSION

### Nucleoside conformation at the mutation site

Wild-type DNA duplexes are defined by isomorphic Watson-Crick (A:T/T:A and G:C/C:G) pairings (WC pairing hereafter), with nucleotides in the *anti* conformation around the glycosidic bond. It is not clear, however, whether or not this conformation will be also favored in mismatched pairs, where isomorphism is lost and WC pairing is not possible. Structural models of mismatched DNA bound to MutS (or MutSα) suggested a *syn* conformation every time a purine is present in the Crick strand (A·A MM: 2WTU (61) and 1OH6 (62); C·A MM: 1OH5 (62), and G·G/G·8oxoG MMs: 1OH7 (62) and 1N2W (63) respectively), while the two MM bases are found in *anti* when thymine are present, as in the Crick strand of the G·T MM (1W7A (64), 1OH8 (62), 1E3M (65), and 2O8B (66)); on the contrary, available NMR structures of naked-DNA with point mutations (T·G (1KKW (67) and 1PIB (68)); G·T (1BJD (69) and 113D (70)); C·T (1FKY (71)), C·C; (1FKZ (71)) and A·G (1ONM (72)) MMs) show both bases are in *anti*, in agreement with the X-ray structure of C·A and A·C pairs (1D99 (70)).

**Figure 2.**
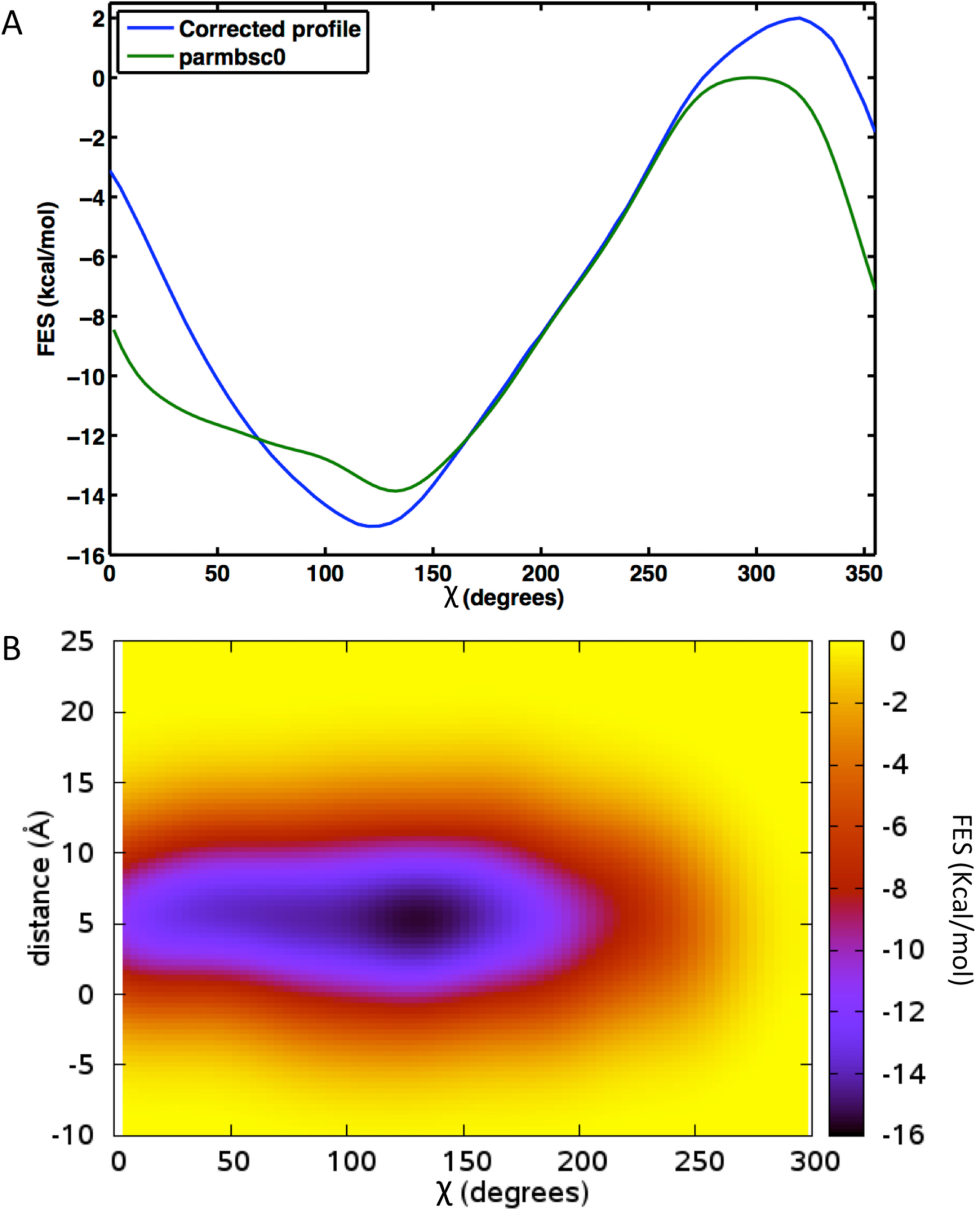
(**A**) Bidimensional free energy profile of G·G MM in the f1 environment as a function of χ angle calculated with WT metadynamics (50,53) (green line), plus the correction (blue line) for potential errors in the representation of glycosidic torsion in parmbsc0 (56) (see methods). (**B**) Tridimensional free energy profile calculated with WT metadynamics (50,53) as a function of the two CVs, χ angle and the G(H1)-G’(O6) HB of Syn conformation. Error in Metadynamics is estimated to be in average 0. 14 kcal/mol.

*Anti→syn* rearrangements are expected to be slower than the sub-microsecond time scale accessible for simulations. Therefore it is unlikely that if *anti/syn* equilibrium exists, it will be well represented during our unbiased trajectories. Taking this into account, it was necessary to start our simulations from the correct conformation. Thus, the first stage in our study was the determination of the most prevalent conformation of the bases at mismatched site. To this purpose, we computed (see Methods) the free energy profile of the *anti←→syn* transition in one of the MMs: the G·G (placed in f1 environment; see below). Note that G·G is a pair known to exist in the *anti-syn* conformation in different protein/DNA complex (73,74), and, accordingly, the system for which the transition to the *syn* conformation is most likely to occur. However, results shown in Figure 2 strongly suggest that the *syn* conformation is disfavored compared to the *anti*, even for the G·G MM. We could then expect a little population of the *syn* conformation in MM DNAs. To corroborate this finding, we collected NMR data for all the Pur·Pur (G·A, A·G, A·A and G·G) MMs (see Methods).

**Figure 3.**
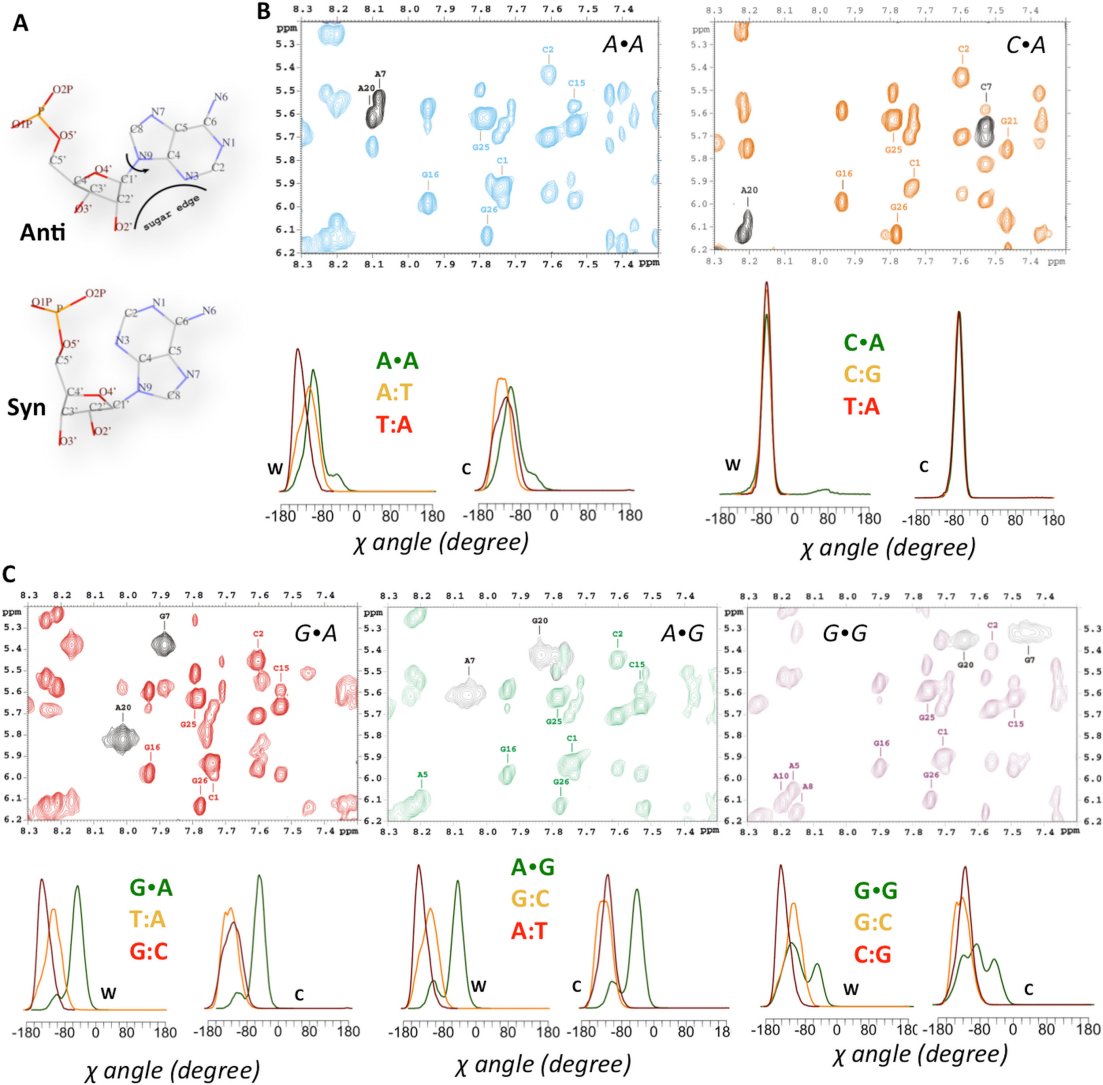
MD and NMR cross-validation of the χ conformation of the MM. (**A**) *Syn*-*anti* scheme; (**B**) and (**C**) NOESY spectra with the correspondent χ distribution for Watson (W) and Crick (C) strands from our simulations for the A·A, C·A, G·A, A·G and G·G duplexes. The H1′-aromatic region of the NOESY spectra is shown with the key intra-residual H1′-H6/H8 cross-peaks highlighted in black color.

NMR spectra confirm the prevalence of the *anti* conformation in all the studied sequences. This is shown in the intensity of intra-residual H1′-H8 NOE cross-peaks in the MM, which is comparable to the other intra-residual H1′-H8 cross-peaks (See Supplementary Table S2 in SI), indicating that all the glycosidic angles are in *anti* (see Figure 3). Interestingly, H1′-H8 cross-peaks appear broaden in G·A, A·G and G·G MMs, suggesting that for these three MMs the *high-anti* conformation is significantly populated, as found in our unbiased MD simulations (see below), as well as suggested by free energy calculations. In addition, recent MD simulations of the DNA-MutS complex with a G·T MM show that both bases are stable and stay in the *anti* conformation during all the simulation (75). Based on these theoretical and experimental results, the MD simulations for the different MMs in the three different environments were started from modeled systems with both nucleotides at the mismatched pair in *anti* conformation.

Note that in any case, no restraints were introduced in these MD trajectories, allowing the system to explore non*-anti* conformations. However, while oscillations in glycosidic torsion were large, especially for Pur·Pur pairs, and *high anti* region was extensively sampled (as also suggested by the NMR spectra; see Figure 3), not a single stable transition to the *syn* states was detected in our more than 7 microsecond accumulated trajectories. Then, unbiased MD simulations, free energy and NMR results strongly suggest that the *syn* conformation, found in some mismatched DNA–protein complexes (76), is directly related to a protein-induced perturbation. The appealing hypothesis that spontaneous population of the *syn* conformation acts as a signal for recruitment of repairing proteins at mismatched site is not likely to be realistic.

### Global structural and energetic changes induced by MMs

**Figure 4.**
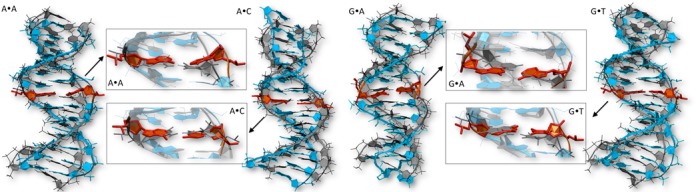
Superimposition of selected MMs (A·A, A·C, G·A, G·T, T·T) over the canonical A:T oligomer in the f1 environment. A close-up in the region of the MM is also shown. The oligomer with the MM is shown in blue ribbon, while the reference WT oligomer is shown in grey. The MM is highlighted in red. The structure used for each system is the main cluster representative over the overall trajectory (see methods for clusterization details).

None of the 36 unrestrained MD simulations performed here with MMs showed massive distortions of the global structure of the duplexes. The root main square deviations (RMSds) between the different trajectories and the average structure of the wild-type duplex(es) are typically around 2.0 Å, with individual values in the range 1.3–3.0 Å (see Supplementary Table S3), not far from the thermal noise of the simulation (in average 1.2 Å). This indicates that the overall impact of MM on the global duplex structure is quite moderate (Figure 4).

The largest global structural changes are typically found for transversions (i.e. pairings Pyr·Pyr or Pur·Pur), but even these dramatic changes, which imply the loss of isomorphism of base steps, do not produce dramatic global alterations in the duplex (Figure 4). Looking at the available structural data of MutS-DNA complexes, a clear global bending is observed in the DNA with a hinge point at the MM position (see Supplementary Figure S3). This particular distortion found after the binding of MutS might be favored by the presence of pre-bent states induced by the MMs before the binding. However, MD simulations suggest that in general this is not the case. Despite few exceptions (like the C·T MM), the helical bends found in our simulations of mismatched DNAs fall within the range (19–25 degrees) detected for control wild-type sequences (see Table 1 and Supplementary Table S4 in SI). This finding agrees with recent umbrella sampling calculations by Feig and coworkers that showed that large bending of either homoduplex or heteroduplex DNA is not a spontaneous process (16). Therefore, in order to achieve bent DNA as observed in the crystal structures in complex with MutS (or MutSα) (61–66), specific DNA–protein interactions are needed. The same conclusion can be reached with respect to global helix twist, which for DNA-containing MMs typically falls (one exception might be the under-twisted A·G MM) within the expected range of variability of the canonical sequences (see Table 1). In summary, irrespective of the MM and surrounding sequences, B-DNA structure seems to be resilient enough as to absorb a significant local perturbation, such as mismatching, without leading to major alterations in the global structure of the helix. This structural-buffer capability surely contributes to maintain DNA functionality despite the presence of lesions.

**Table 1.** Global helical parameters averaged overall the sequence for each MMs and canonical systems

	Helical bend (degrees)	Breathing (%)	minW (Å)	Helical Twist (degrees)
A:T	23.46±3.69	1.74±0.47	6.26±0.11	316.60±10.04
C:G	24.97±0.24	0.15±0.05	6.58±0.09	309.73±6.10
G:C	23.56±3.71	0.08±0.02	6.75±0.09	323.39±0.97
T:A	19.42±0.08	2.90±0.73	6.13±0.08	324.24±2.56
A·A	22.58±0.63	40.15±15.29	7.41±0.06	316.30±4.52
A·C	27.30±0.13	22.00±3.80	6.91±0.08	317.24±6.56
A·G	25.67±0.29	18.90±19.73	8.14±0.51	297.90±3.91
C·A	22.19±1.16	23.33±6.04	6.79±0.12	317.49±5.88
C·C	23.78±2.75	1.18±0.13	4.95±0.02	323.70±2.45
C·T	30.91±1.42	10.54±5.29	4.97±0.08	305.42±13.94
G·A	22.88±1.31	12.08±2.71	8.20±0.13	315.65±4.92
G·G	24.79±4.32	66.95±14.42	7.61±0.28	306.71±9.92
G·T	19.02±0.77	14.16±5.15	6.91±0.09	324.99±0.80
T·C	25.00±0.06	5.06±1.49	4.78±0.00	325.17±5.63
T·G	18.25±0.30	10.87±1.85	6.54±0.23	323.39±1.64
T·T	18.59±1.19	18.99±3.04	5.76±0.13	325.74±1.24

The corresponding sequence-dependent global helical parameters are reported in Supplementary Table S4.

### Local structural changes induced by MMs

**Figure 5.**
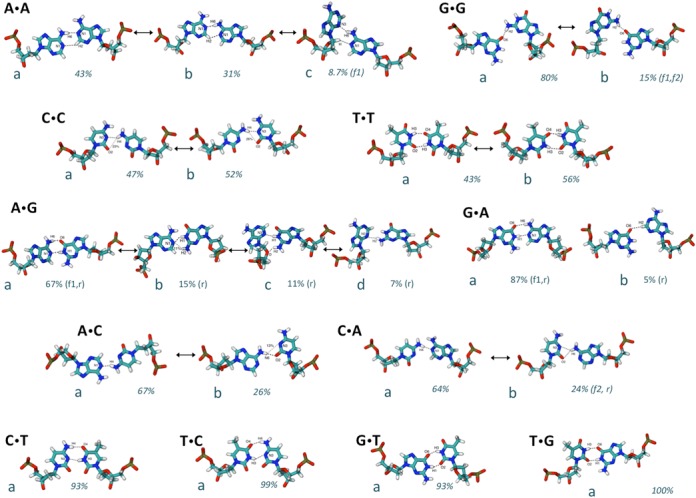
The scheme of HB pairings in A·X, C·X, G·X and T·X MMs visited during our simulations. The percentage of occurrence of each pairs is also indicated. When the environment is explicitly mentioned in parenthesis, it means that the HB-scheme occurs only in that environment.

The presence of transversions or transitions MM does not dramatically modify the overall helix geometry, but has a significant impact in the energetics of base-pair interactions (Supplementary Figures S4–S6) and accordingly in local base step geometry, especially (but not only) at the mutation site. Contrary to the situation in the wild-type sequences, where the WC pairing scheme is very well preserved in all the trajectories, hydrogen-bonding (H-bonding) patterns in MMs are often labile, and in some cases promiscuous. Analysis of the 12 × 3 trajectories generated here shows different degrees of H-bonding promiscuity in the different pairs. Thus, Pyr·Pyr transversions (see Figure 5) show in general only one dominant pairing scheme (which obviously in homopyrimidine pairs lead to two mostly equally populated, symmetric dimers). On the contrary, Pur·Pur transversions populate many different pairing schemes, as a result of large rotation and translations of one base with respect to the other (see Figure 5 and Supplementary Figure S7). These movements allow the bases to explore not only interactions through the WC side, but also through the major and minor grooves, which in some cases involve interactions with the sugar atoms typically found in tridimensional RNA structures (see Figure 5). Finally, the situation for Pur·Pyr transductions is somehow intermediate, for the stable MMs G·T and T·G. Specifically a single H-bonding pattern is present in more than 93% of the collected snapshots, while for A·C and C·A pairs two H-bonding schemes, related by simple in-plane rotations of the two bases coexist (see Figure 5). Despite the different H-bonding schemes, in almost all the cases, the preferred H-bonding scheme position actually coincides with the canonical one (see Supplementary Table S5). The scenario of H-bonding schemes observed during our simulations is then richer than that previously suggested in smaller-scale studies of mismatched B-DNA duplexes (77).

The promiscuity of HB scheme and the mobility of base pairs generate very large changes in the orientation of bases around the glycosidic torsion (see Supplementary Figure S8), and in the average intra-base pair helical parameters (particularly stretch, shear and opening; see Supplementary Figure S9 and Table S6), as well as widening in the equilibrium distributions in many cases (for example G·G). As a result, a dramatic increase in the frequency and magnitude of breathing becomes evident. Thus, while we estimate breathing to affect only between <0.2% (G:C) and < 3% (A:T) of collected snapshots in the wild-type duplexes, mismatched pairs can yield to very significant breathing populations. Some base pairs seem more affected by breathing than others, for example, C·C breathing propensity is close to that of a canonical pair (around 1%), while pairs such as G·G and A·A show breathing frequencies above 40% (see Table 1). To better characterize such behavior, we calculated the time evolution of the breathing events (Supplementary Figure S10), as well as the average residence times, occupancies and number of transitions, discriminating between the major and the minor groove (Supplementary Table S7), for three selected cases showing low, moderate and high breathing respect to a canonical Watson-Crick pairing. As expected, the mismatched bases prefer to breathe toward the major groove, showing a great variability in frequency and duration of the breathing events, which is particularly noticeable across the different type of mismatches.

**Figure 6.**
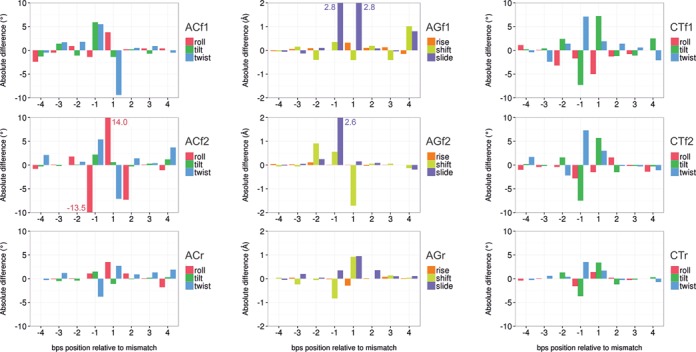
*Lesion information transfer*. Structural distortions induced by the MMs on the neighboring base pairs steps (bps) for selected MM in different environment (f1, f2, r, see Figure 1). The absolute difference was obtained by subtracting the average value of a given helical parameters and a given bps in the MM simulation to the same helical parameter and bps in the canonical simulations. Note that the sequence is referred relative to the MM base pair (position 0). Translational (angstroms) and rotational (degrees) helical parameters are displayed separately. The Lesion information transfer for all the other MMs are reported in SI, Figures S11–S13.

**Figure 7.**
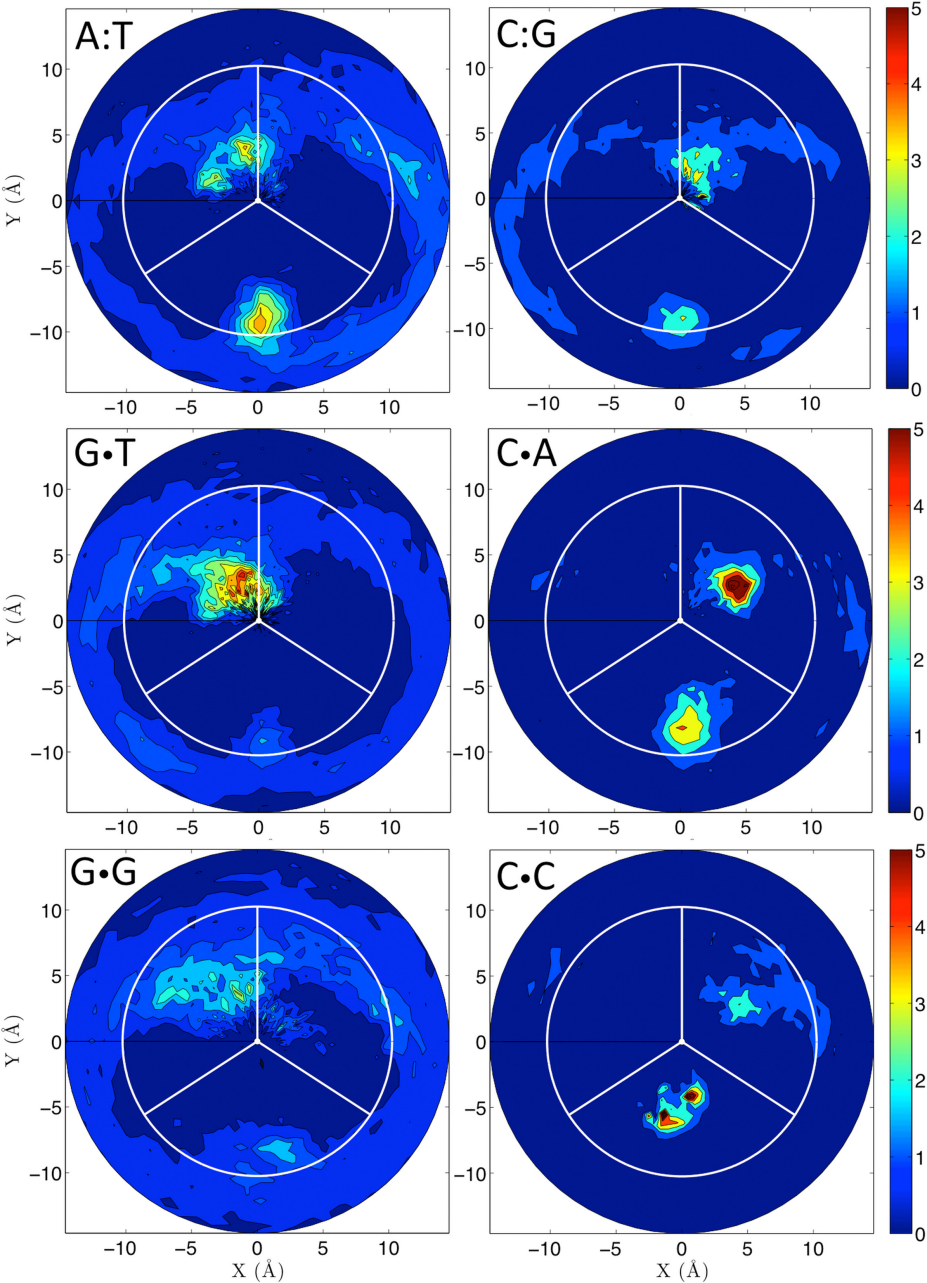
*Distortion of the ionic atmosphere*. Two-dimensional cation distributions averaged over the last 100 ns of the trajectories. The plot show the radial-angular plane at the central MM base pair, the minor groove limits as white lines and the center of the major groove as a vertical radial vector. The results are plotted as molarities as shown by the color bars, with a blue to red concentration scale that goes from 0 to 5 molar. The two canonical base pairs (A:T, C:G) and four selected MMs in the flexible environment are shown.

The presence of the MMs not only introduces changes in intra base-pair helical parameters, but also in inter base-pair helical parameters, which are especially large in Pur·Pur transversions (see Supplementary Table S8). In general, all the MMs lead to local increase of roll and typical compensatory up-down changes in tilt, while the local changes in twist depend on the nature of the MM. Thus, while the Pur·Pur ones lead to under-twisted steps, Pyr·Pyr pairs produce overtwisted steps and Pur·Pyr pairs display compensatory changes (at the 3′ and 5′ sides of the lesion; see Figure 6 and Supplementary Figures S11–S13) affecting not only the depth (see above), but also the width of the grooves, particularly of the minor groove (Table 1). Except for T·T pairings, where signal is less clear, Pyr·Pyr pairings lead to a clear compression of the minor grove, while Pur·Pur pairs lead to the opposite effect (Table 1). Not surprisingly, these groove perturbations induced significant alterations in the ion environment around the DNA (see examples in Figure 7). Notably, while some MMs clearly alter the groove dimensions and the capability to bind cations (GG, CA and CC), the ion cloud around the GT transduction is again very similar to the canonical AT (Figure 7).

The alteration of the ion environment, in turn may affect the ability of the DNA to be recognized by external partner, like also our calculation of classical molecular interaction potential at the DNA groove seems to suggest (see examples in Supplementary Figure S14).

It is difficult, probably impossible, to find simple relationships between the distortions induced by MM and the ability of the lesion to be recognized and repaired by MutS (or its homologous). The reasons are multiple: (i) there is no consensus ranking of MutS susceptibility of the different MMs, and (ii) analysis of available X-Ray complexes of mismatched DNA bound to MutS (or MutSα) (PDB ids: 1W7A (64), 2WTU (61), 1OH5 (62), 1OH6 (62), 1OH7 (62), 1OH8 (62) and 2O8B (66)) suggests that not only indirect, but also direct recognition mechanisms play a role in defining MutS preferences. However, some general trends become evident when comparing consensus MutS recognition data with MM-driven structural/dynamical distortions. For example, Pur·Pur MMs are generally better recognized than the Pyr·Pyr ones (20–22,78), which agree with the high frequency of breathing and with the wider minor groove (see Table 1), two factors that should favor protein–DNA recognition of the Pur·Pur pairs compared with Pyr·Pyr. Not surprisingly, G·G and A·A (where breathing frequency is 40–67%, and average minor groove width is 1 Å wider than the wild type), are among the best-recognized and repaired MMs (20–22,78), while G·A pair (low breathing frequency) is the worst recognized Pur·Pur MM (Table 1). Similar considerations helped us to understand why the C·C MM is very poorly recognized (it displays simultaneously a very low breathing frequency and a very narrow minor groove), while the T·T MM that does not show a wider minor groove width but has 20% of breathing is reasonably recognized and repaired. Even though specific details of protein–DNA interaction are surely crucial to modulate the recognition of lesions by MutS, local changes induced by the presence of the MM on the structure and dynamics of DNA, certainly help the enzyme to discriminate between canonical and mismatched DNAs.

### Lesion information transfer

As described above the structural changes induced by MMs do not lead to global changes detectable in the global properties of DNA. However, this does not necessarily mean that the geometry of remote base steps is not affected by the presence of mismatched pairs. Local analysis along the helix confirms that MMs indeed introduced local perturbations that are not always confined to the lesion step, but can be transferred to remote locations (see Figure 6 and Suppl. Supplementary Figures S11–S13). The mode and magnitude of the lesion information transfer depend on the nature of the MM and especially on the flexibility of the environment (more flexible sequences transfer better distortion than the rigid one). Nevertheless, magnitude of the perturbations is significant when compared to the standard error, and the probability of observing such structural deviation out of the distributions of the control oligomers. For instance, in the ACf2 mismatch, we observed an average deviation of 3.7 degrees on the twist angle at the bps 4 with respect the same bps in the canonical simulations (Figure 6). The standard deviation for the twist in the mismatch simulation for this specific bps is 4.6 degrees, with a standard error of 0.03 degrees. Using the distribution of the control simulation, for the same bps, the probability of getting a single structure with a twist deviation of 3.7 degrees is only 6%. In the same way, we analyzed the deviation in the shift helical parameter observed at bps 4 for the AGf1 mismatch. In this case, we observe a deviation of 1.01 Å, the standard deviation in the MM simulation for this bps is 0.63 Å, the standard error >0.01 Å, and the probability of getting a single structure with a shift deviated 1.01 Å from the average of the canonical distribution is 17%. Additionally, a *t*-test was used to test the null hypothesis of no differences in the mean values of the distributions at bps 4 made by the presence of the mismatch. In both cases (twist in ACf2 and shift in AGf1) the null hypothesis was rejected with a *p*-value of 0.01. In this way, the deviations found in the mean values (or similarly, the shift between both distributions), half-turn away from the mismatch site, turns to be truly significant.

It is worth noting that a significant part of the perturbation is transferred through the backbone by means of typically anti-correlated movements (e.g. over-twist in one step often leads to under-twist in the contiguous one), but there seems to be a previously unknown (to our knowledge) through-space mechanism, which is noticeable at $\frac{1}{2}$ DNA helical turn away from the mismatch site (see Figure 6 at positions −4 and 4).

The basic idea that a perturbation produced to the DNA at position X, can travel several base pair and affect the behavior of the DNA at position Y, is not completely new. This effect was recently demonstrated to occur experimentally (79) and to be relevant to explain the cooperative binding of two proteins to the DNA. Nevertheless, for all the studied cases, the very low strength of the signal was a common feature. Until the Science 2013 publication (79), DNA allostery was elusive for the experimental setups, since its characteristic signal occurs slightly above the thermal noise. In this work, we hypothesized that information transfer can also occurs through the environment surrounding the DNA. As shown in the previous paragraph, we can expect that this kind of information transfer also display a very low signal, difficult to separate from the thermal noise and, consequently, difficult to understand. The mechanism of information transfer is not clear, since correlations between backbone torsions are weak (typically below 0.2). The fact that information could travel half-turn away from the MM, suggests a through-space transfer mechanism mediated by changes in hydration and ion atmosphere in the groove, which are connected with conformational transitions between substates in DNA (80,81). We believe that the perturbed environment around the DNA at the mismatch site could produce a change on the DNA behavior, acting as a vehicle for spreading the signal of the damage half-turn away from the lesion. The effect of such information transfer could likely be an ‘antenna’, contributing to amplification of the lesion signal and favoring recruitment of repairing proteins.

## CONCLUSION

The global structure of DNA is extremely plastic, as being able to absorb the distortion introduced by MMs without altering its overall helical shape. However, at the local level, the impact of MMs can be from moderate to very extended, depending on the nature of the MM. The concept of ‘mismatched DNA’ should be then avoided, since in some cases, like C·C, structural and dynamic distortions are mild, while in others, such as G·G, they are very severe. Changes in the dimensions of the grooves, particularly the minor one, and a higher frequency of breathing (related often to the promiscuity of H-bonding schemes and visits to the high-anti region of the glycosidic bond) are the most common MM-induced changes irrespectively of the sequence environment. However, the spontaneous transition to the *syn* conformation is not supported by our unbiased calculations and is ruled out by biased MD simulations and by high-resolution NMR experiments.

The capacity of DNA to buffer distortion is extremely large, which probably explains the robustness of this molecule. Mechanistically, in some cases (depending on the sequence) relaxation of the perturbation occurs by a compensatory through-backbone and through-space transfer mechanisms, which can transfer signals of lesions to remote locations. As chemical intuition suggest, flexible sequences transfer better the lesion information than the rigid ones, which can confine the lesion to the MM site. This sequence-dependence in distortion transfer can explain why the same MM embedded in different sequence environments can be recognized and repaired with quite different efficiency by MutS, and can generate an ‘antenna effect’ for recruitment of repairing proteins.

It is impossible to connect in an unambiguous way the nature of structural distortion induced by the MM and the efficiency in which this lesion is recognized by MutS (or the homologous in human, MutSα (19)), since this process is expected to use both indirect and direct reading mechanisms. However, clear connections are found between the nature of the MM-induced perturbation and the ability of MutS (or MutSα) to recognize such lesions. We found that several of the best-recognized MMs display normal to wider minor grooves and high frequency of breathing, which leads to the partial occupancy of the grooves by the bases, while poorly repaired MMs display low frequency of breathing and in general narrow minor groove that will not accommodate well MutS (or MutSα) active residues. Thus, indirect reading mechanisms related to the structural and dynamic distortion induced by MM clearly contribute in the recognition of the damaged DNA by means of the key repairing enzyme MutS and its homologues.

## SUPPLEMENTARY DATA

Supplementary Data are available at NAR Online.

SUPPLEMENTARY DATA
