# Supplementary material for: The structural impact of DNA mismatches
Source: Nucleic Acids Res. 2015 Mar 27;43(8):4309–21. doi: 10.1093/nar/gkv254 (PMC4417165; doi:10.1093/nar/gkv254)
Supplement: SUPPLEMENTARY DATA [file supp_43_8_4309__index.html]

The structural impact of DNA mismatches — SUPPLEMENTARY DATA 

# The structural impact of DNA mismatches

## SUPPLEMENTARY DATA

**Files in this Data Supplement:**

- SUPPLEMENTARY DATA
