# Supplementary material for: The structural impact of DNA mismatches
Source: Nucleic Acids Res. 2015 Mar 27;43(8):4309–21. doi: 10.1093/nar/gkv254 (PMC4417165; doi:10.1093/nar/gkv254)
Supplement: SUPPLEMENTARY DATA [file supp_gkv254_nar-03644-f-2014-File009.pdf]

The structural impact of DNA mismatches

Giulia Rossetti[1,2,3,6], Pablo D. Dans[1,6], Irene Gomez-Pinto[1,4], Ivan Ivani[1], Carlos Gonzalez[4] and Modesto Orozco[1,5,*]

[1] Joint BSC-CRG-IRB Program on Computational Biology, Institute for Research in Biomedicine (IRB Barcelona), Baldiri Reixac, 10, Barcelona 08028, Spain.
[2] Computational Biophysics, German Research School for Simulation Sciences (Joint venture of RWTH Aachen University and Forschungszentrum Jülich, Germany), D-52425 Jülich, Germany and Institute for Advanced Simulation IAS-5, Computational Biomedicine, Forschungszentrum Jülich, D-52425 Jülich, Germany.
[3] Juelich Supercomputing Center (JSC), Forschungszentrum Jülich, Jülich, Germany.
[4] Instituto de Química Física Rocasolano. CSIC. C/Serrano 119. Madrid 28006. Spain
[5] Departament de Bioquímica i Biologia Molecular. Facultat de Biologia. Universitat de Barcelona. Avgda Diagonal 647. Barcelona 08028. Spain.
[6] The authors wish it to be known that, in their opinion, the first two authors should be regarded as joint First Authors
* To whom correspondence should be addressed. Tel: +34 93 40 37156; Fax: +34 934 037 157; Email: modesto.orozco@irbbarcelona.org
Present Address: [Giulia Rossetti], Computational Biomedicine, Institute for Advanced Simulation IAS-5, Institute of Neuroscience and Medicine INM-9, and Jülich Supercomputing Centre, Forschungszentrum Jülich, 52425 Jülich, Germany

Supporting Information

Trajectory analyses.

Structural analysis was performed using standard tools in our NaFlex server(1), Gromacs 4.5.5 toolkit(2), and AmberTools14(3). DNA helical parameters and backbone torsion angles associated with the mismatched base pair and its neighboring steps were measured with the Curves+ and Canal programs(4,5) without considering capping bas pairs. The new module from Curves+(6) was used to determine the position of each cation in curvilinear cylindrical coordinates for each snapshot of the simulations (last 100 ns) with respect to the instantaneous helical axis. Ion distributions were computed for the two base pair steps flanking the lesion (*D* = 5.8 to 8.2) inside the grooves (R = 10.25 Å), and dividing the contribution between the minor groove (*A* = 33º to 147º) and the major groove. As in our previous work(7), two-dimensional radial-angular (*RA*) plots were obtained representing ion densities in units of molarity(6). Flexibility analysis was performed using standard protocols described elsewhere(8,9). Our classical molecular interaction potential(10) was used to analyze the ability of DNA to recognize sodium ions. The electrostatic interaction term was determined by solving the linear Poisson–Boltzmann equation(11), while the van der Waals contribution was determined using standard AMBER Lennard–Jones parameters(10,12). The ionic strength and the reaction-field dielectric constant were set to 0.15 and 78.4 M, respectively, while the dielectric constant for DNA was set to 8(13). The calculations were performed using the same average structure used to extract the helical axis for the cation analysis previously described. The frequency of DNA breathing was computed only for the MM base pair, based on the opening parameter measured with Curves+ package(4,5). We consider that the base pair was "breathing" when the opening value deviated more than ±20 degrees from the canonical B-value (-3.6 degrees). The frequency was finally expressed as the number of occurrences of breathing per ns. The number of HBs were calculated at the base pairs -1;0+1 with the tool g_hbond of Gromacs 4.5.5 toolkit(2). Stacking energies were calculated by considering Coulomb

and Lennard-Jones contributions for each couple of stacking bases. Cross terms (CT) energies were calculated by considering Coulomb and Lennard-Jones contributions between the base i_W in the Watson (W) strand with the bases $(i+1)_C$ and $(i-1)_C$ in the Crick (C) strand and between i_C and the bases $(i+1)_W$ and $(i-1)_W$. Hydrogen bond (HB) energies were calculated by considering Coulomb and Lennard-Jones contributions between base pairs. For all the energetic calculations the module g_energy of Gromacs 4.5.5 toolkit(2) was used. Additional non-standard analysis were carried out on-purpose with R-scripts developed in our group(14). The trajectories were clustered with the GROMOS method(15), implemented in the g_cluster module of Gromacs 4.5.5 toolkit(2), using an RMSD cutoff of 10 Å.

Table S1. Stiffness constants associated to global (helical axis bend), semi-global (minor groove width), and local (opening) deformations of 13 base pair oligomers with sequences d(CCATACXATACGG) (f1), d(CCAATTAXAATTGG) (f2), and d(CCCAGTXCTTTGG) (r), where X is one of the four possible canonical base pairs (A:T, T:A, C:G and G:C). Note that sequence averaged stiffness constants are highlighted in bold. See Figure 1 for details about the chosen sequences.

Helical axis bend (kcal/mol·deg^2)[a]					
ATf1	0.224	ATf2	0.108	ATr	0.226
CGf1	0.180	CGf2	0.277	CGr	0.505
GCf1	0.302	GCf2	0.247	GCr	0.634
TAf1	0.183	TAf2	0.328	TAr	0.263
	**0.222**		**0.240**		**0.407**
Minor groove width (kcal/mol·Å^2)[b]					
ATf1	0.084	ATf2	0.031	ATr	0.097
CGf1	0.122	CGf2	0.158	CGr	0.248
GCf1	0.163	GCf2	0.138	GCr	0.402
TAf1	0.092	TAf2	0.075	TAr	0.101
	**0.115**		**0.100**		**0.212**
Opening (kcal/mol·deg^2)[c]					
ATf1	0.148	TAf2	0.059	ATr	0.175
TAf1	0.161	ATf2	0.147	TAr	0.187
GCf1	0.172	CGf2	0.195	CGr	0.494
CGf1	0.312	GCf2	0.216	GCr	0.614
	**0.198**		**0.154**		**0.368**

[a] The helical bend was computed between base pair steps 2 and 11 using the *sumr* tool from the curves+ package (4,5). [b] The minor groove width was taken as an average of the values computed with curves+ for base pairs numbers 6, 7 and 8. [c] Only the opening of the central base pair (base pair number 7) was considered.

Table S2: Intra-residual H1'-H8 distances obtained from a complete relaxation matrix analysis of experimental NOE intensities. Lower and upper distance limits result from error analysis of several calculations considering different structures, correlations times and NOESY mixing times (see Mat and Methods section).

**H1'-H6**	**AAf**	**CAf**	**GAf**	**AGf**	**GGf**
**C1**	---	---	---	---	---
**C2**	2.82..4.23	3.08..4.02	2.36..4.15	3.57..4.07	3.34..3.91

**A3**	2.89..3.59	2.60..3.40	3.17..3.94	3.37..3.87	3.30..3.80
**T4**	2.69..4.19	2.86..3.76	2.90..4.20	3.27..4.01	3.37..3.97
**A5**	2.92..3.62	---	3.02..4.92	3.34..3.84	3.00..4.00
**C6**	4.05..4.67	3.06..3.86	---	---	---
**X7**	2.80...4.00	---	3.25..4.37	3.77..4.57	3.04...4.54
**A8**	4.15..4.74	3.79..4.92	3.66..4.66	---	2.81..3.81
**T9**	2.69..3.59	2.98..3.48	3.34..4.14	3.86..4.36	3.81..4.31
**A10**	3.30..3.80	--	2.70..3.70	---	3.48..3.98
**C11**	3.17..4.79	2.56..3.96	2.93..3.86	3.75..4.75	3.63..4.13
**G12**	---	---	---	---	---
**G13**	---	---	---	---	---
**C14**	2.16..4.16	3.04..4.64	3.51..4.21	3.51..4.21	2.00..4.00
**C15**	3.00..3.70	2.73..4.32	2.93..3.53	3.73..4.73	3.61..4.11
**G16**	2.67..3.77	2.88..4.01	3.12..4.02	3.65..4.03	2.80..3.40
**T17**	2.25..4.23	2.77..4.27	2.81..3.99	3.27..4.27	---
**A18**	2.97..3.67	2.66..3.99	---	---	---
**T19**	3.27..3.77	---	3.23..4.63	3.04..3.77	3.77..4.41
**X20**	3.50..4.61	3.64..4.44	3.20..4.60	2.50..4.50	3.53..4.53
**G21**	---	3.65..4.15	2.80..3.96	---	---
**T22**	---	---	2.81..4.00	3.57..4.27	---
**A23**	3.24..3.98	2.95..3.92	2.86..3.86	---	---
**T24**	2.58..4.08	3.17..3.67	3.12..3.62	3.63..4.13	3.73..4.23
**G25**	---	---	---	---	---
**G26**	---	---	---	---	---

---: NOE intensity cannot be determined due to cross-peak overlapping.

Table S3. Average RMSd values over the simulated time and Standard Deviation (SD) for each MMs with respect the correspondent canonical DNA. Capping Base Pairs and the MM base pair were not considered.

**MM**	**RMSD (Å)**	**SD (Å)**	**MM**	**RMSD (Å)**	**SD (Å)**
**A·Af1**	1,61	0,53	GAf1	3,02	0,44
**A·Ar**	1,31	0,31	GAr	2,23	0,21
**A·Af2**	1,63	0,48	GAf2	2,33	0,28
**A·Cf1**	1,89	0,23	GGf1	2,70	0,47
**A·Cr**	1,82	0,25	GGr	2,57	0,33
**A·Cf2**	1,79	0,23	GGf2	2,42	0,21
**A·Gf1**	2,79	0,60	GTf1	2,23	0,18
**A·Gr**	1,66	0,26	GTr	2,19	0,17
**A·Gf2**	2,00	0,41	GTf2	2,23	0,20
**C·Af1**	2,62	0,24	TCf1	1,91	0,23
**C·Ar**	2,54	0,19	TCr	1,82	0,21
**C·Af2**	2,58	0,19	TCf2	1,80	0,17
**C·Cf1**	2,81	0,20	TGf1	1,71	0,24
**C·Cr**	2,74	0,15	TGr	1,69	0,25
**C·Cf2**	2,81	0,17	TGf2	1,74	0,28
**C·Tf1**	2,57	0,16	TTf1	1,30	0,29
**C·Tr**	2,54	0,17	TTr	1,30	0,31
**C·Tf2**	2,52	0,13	TTf2	1,36	0,30

Table S4. Global helical parameters

System	Helical bend[1] (degrees)			Breathing[2] (%)	n HB[3]			minW[1] (Å)			Helical Twist[1] (degrees)			Helical Rise[1] (degrees)		
ATf1	22.3	±	11.4	1.2	7.2	±	0.7	6.5	±	1.1	326.3	±	11.2	31.8	±	1.2
ATr	18.3	±	9.3	1.5	7.1	±	0.7	6.3	±	1.0	324.3	±	10.6	32.2	±	1.0
ATf2	29.9	±	19.3	2.6	5.9	±	0.6	6.1	±	1.2	299.2	±	34.6	33.3	±	1.2
CGf1	31.5	±	17.5	0.2	8.3	±	0.8	7.0	±	0.9	310.7	±	41.0	31.8	±	1.7
CGr	18.1	±	9.8	0.1	8.3	±	0.8	6.0	±	1.1	319.3	±	12.1	32.3	±	1.0
CGf2	25.4	±	14.6	0.1	7.2	±	0.7	6.7	±	1.0	299.2	±	25.2	33.0	±	1.0
GCf1	23.2	±	12.1	0.1	8.3	±	0.8	6.7	±	1.1	326.4	±	11.0	31.8	±	1.1
GCr	17.5	±	9.1	0.0	8.3	±	0.8	6.7	±	1.0	322.0	±	10.8	32.2	±	1.0
GCf2	30.0	±	15.8	0.1	7.2	±	0.7	6.9	±	0.9	321.7	±	24.2	32.2	±	1.3
TAf1	18.9	±	10.1	4.2	7.1	±	0.7	6.7	±	1.1	321.3	±	10.2	31.3	±	1.1
TAr	19.8	±	10.4	1.4	7.1	±	0.7	5.4	±	1.1	322.8	±	13.5	32.4	±	0.9
TAf2	19.6	±	10.4	3.2	6.0	±	0.5	6.3	±	1.1	328.7	±	24.3	32.6	±	0.8
AAf1	29.2	±	14.9	33.0	6.0	±	0.8	7.7	±	1.4	300.7	±	27.0	31.9	±	1.6
AAr	17.0	±	9.2	20.8	6.0	±	0.8	7.2	±	1.0	324.1	±	12.9	32.2	±	0.9
AAf2	21.5	±	10.6	66.6	4.4	±	0.8	7.3	±	1.4	324.1	±	20.5	33.1	±	1.1
ACf1	30.6	±	14.0	11.6	6.3	±	0.9	7.0	±	1.1	296.6	±	16.1	32.3	±	1.2
ACr	24.2	±	11.6	28.6	6.2	±	0.9	7.0	±	1.2	326.5	±	10.4	32.1	±	1.0
ACf2	27.1	±	14.0	25.8	5.0	±	0.8	6.8	±	1.1	328.6	±	12.8	32.6	±	0.9
AGf1	33.9	±	18.0	2.6	7.3	±	0.9	9.6	±	1.0	264.8	±	26.4	33.2	±	1.5
AGr	17.0	±	9.2	1.0	7.7	±	1.0	7.6	±	0.9	324.2	±	10.6	32.1	±	0.9
AGf2	26.2	±	16.1	53.1	5.5	±	1.2	7.3	±	1.4	304.7	±	27.5	33.4	±	1.5
CAf1	24.9	±	13.4	30.4	6.3	±	0.9	7.1	±	1.3	327.2	±	22.3	31.8	±	1.2
CAr	21.5	±	15.1	5.8	6.3	±	0.9	6.7	±	1.2	297.6	±	88.3	31.7	±	2.1
CAf2	20.2	±	10.4	33.8	4.8	±	0.8	6.6	±	1.2	327.7	±	16.7	32.6	±	0.8
CCf1	24.0	±	13.2	1.1	6.2	±	0.9	5.1	±	1.3	321.2	±	18.5	32.9	±	1.3
CCr	18.8	±	10.7	1.1	6.5	±	0.9	4.9	±	1.1	330.4	±	16.6	32.4	±	0.8
CCf2	28.5	±	18.5	1.4	5.1	±	0.8	4.9	±	1.1	319.5	±	32.3	32.5	±	1.1
CTf1	35.9	±	17.5	3.5	7.1	±	0.7	4.5	±	0.8	270.6	±	120.2	32.3	±	1.2
CTr	23.5	±	13.4	19.7	6.6	±	1.1	5.6	±	1.3	316.1	±	23.6	32.6	±	1.0
CTf2	33.4	±	15.1	8.4	5.9	±	0.7	4.8	±	1.0	329.6	±	46.2	32.8	±	1.1
GAf1	30.9	±	16.4	4.1	7.4	±	0.9	9.2	±	1.3	300.1	±	16.8	32.4	±	1.6
GAr	17.1	±	9.3	16.8	7.2	±	1.3	7.4	±	1.0	322.7	±	12.5	32.1	±	0.9
GAf2	20.6	±	11.7	15.3	6.1	±	1.1	8.0	±	1.2	324.2	±	13.9	32.6	±	0.9
GGf1	27.2	±	13.9	64.6	5.8	±	0.9	7.8	±	1.9	314.8	±	18.4	32.2	±	1.5
GGr	29.8	±	15.4	44.3	6.1	±	0.9	7.9	±	1.5	281.5	±	105.6	32.6	±	1.6

GGf2	17.3	±	9.7	91.9	4.8	±	1.0	7.1	±	1.2	323.9	±	15.2	32.7	±	0.9
GTf1	19.1	±	10.1	23.1	7.2	±	0.8	6.8	±	1.1	324.9	±	15.1	31.5	±	1.2
GTr	17.7	±	10.7	6.2	7.2	±	0.9	6.9	±	0.9	323.7	±	13.4	32.0	±	1.0
GTf2	20.4	±	9.9	13.2	6.2	±	0.7	7.1	±	1.0	326.4	±	12.5	32.5	±	0.9
TCf1	26.8	±	13.0	4.6	7.1	±	0.7	4.8	±	1.1	331.7	±	19.7	32.2	±	1.0
TCr	23.4	±	12.0	3.0	7.2	±	0.7	4.8	±	1.1	328.4	±	15.9	32.6	±	0.9
TCf2	24.9	±	14.2	7.6	5.9	±	0.7	4.8	±	1.0	315.4	±	26.7	33.0	±	0.9
TGf1	20.1	±	10.5	14.1	7.3	±	0.8	6.5	±	1.2	323.4	±	12.7	31.4	±	1.2
TGr	16.9	±	9.1	5.1	7.4	±	0.8	6.2	±	1.2	320.6	±	11.2	32.2	±	1.0
TGf2	17.7	±	9.9	13.5	6.2	±	0.7	6.9	±	1.0	326.2	±	13.0	32.5	±	0.9
TTf1	18.0	±	9.4	23.4	7.0	±	0.7	5.8	±	1.0	326.4	±	13.1	31.7	±	1.1
TTr	21.2	±	10.4	9.3	7.1	±	0.7	5.9	±	1.0	322.9	±	11.8	32.4	±	1.0
TTf2	16.5	±	8.5	24.3	5.9	±	0.5	5.5	±	1.0	327.9	±	13.9	32.8	±	0.8

[1]without capping base pairs; [2]At the location of the MM; [3]at base pairs -1;0+1;

Table S5. MM base pair position. The symbol "-", "m" or "M" is used if the MM bases are close to their canonical positions, displaced into the minor, or major grooves respectively. The corresponding HB configuration (Fig. 5 Main Text) is indicated in parenthesis.

MM	configurations	MM	configurations	MM	configurations
**AA**	-(a); M(b); m(c)	**AC**	- (a); M (b)	**AG**	m(a); M(b); M(c)
**CA**	-(a); M(b)	**CC**	-(a); M(b)	**CT**	-(a)
**GA**	-/m(a); M(b)	**GG**	m(a); M(b)	**GT**	-(a)
**TC**	-(a)	**TG**	-(a)	**TT**	-(a); -(b)

Table S6. Average values of the six intra-strand base pair parameters.[a]

Step	Shear	Stagger	Stretch	Buckle	Opening	Propeller
AT	0.05<br>*0.10*	0.06<br>*0.07*	0.04<br>*0.03*	2.1<br>*3.7*	5.7<br>*3.1*	-22.6<br>*-12.9*
GC	0.01<br>*0.02*	0.14<br>*0.10*	0.03<br>*0.03*	0.5<br>*2.8*	1.4<br>*1.1*	-11.1<br>*-9.1*

[a] Values for simulations were obtained from time averages computed for individual steps in each sequence. The values are compared with a large ensemble of MD simulations performed with parmbsc0 (italic), taken from a previous work (Lavery.NAR.2010). Rotational parameters are in degrees (Buckle, Opening, Propeller), and translational ones (Shear, Stagger, Stretch) in Å.

Table S7. Dynamics of breathing events for the major and the minor groove in selected MMs.

	Breathing towards the major groove			Breathing towards the minor groove		
	Avg. Residence time (ps)	Occupancy (%)	Nº of transitions	Avg. Residence time (ps)	Occupancy (%)	Nº of transitions
GGf1	51.9	59.1	227	1.7	5.5	646
GGf2	92.4	91.5	197	1.5	0.4	53
GGf2*	49.0	77.2	1101[a]	2.0	2.3	822[a]
GGr	25.2	35.9	285	2.2	8.4	775
AAf1	4.6	31.3	1371	1.4	1.7	246
AAf2	11.3	64.5	1099	1.4	0.8	112
AAf2*	7.1	54.8	5361[a]	1.5	0.8	339[a]
AAr	5.8	19.4	669	1.4	1.4	207
GTf1	5.7	23.1	814	1.0	>0.1	6
GTf2	2.8	13.1	943	2.0	>0.1	3
GTr	2.7	6.1	460	2.3	0.1	8
C:Gf1	3.4	0.2	15	0.0	0.0	0.0
C:Gf2	1.3	0.1	19	0.0	0.0	0.0
C:Gr	2.4	0.1	10	0.0	0.0	0.0

[*] From extended (700ns) molecular dynamics simulations. [a] The number of transitions are computed over 70000 structures (instead of 20000).

Table S8. Average values of the six inter-strand base pair parameters for the bps presents in the studied sequences.[a]

Step	Rise	Shift	Slide	Roll	Tilt	Twist
AA	3.3<br>*3.3*	-0.4<br>*-0.3*	-0.3<br>*-0.3*	0.4<br>*0.3*	-3.0<br>*-2.6*	35.3<br>*35.4*
AC	3.4<br>*3.3*	-0.1<br>*0.1*	-0.8<br>*-0.6*	-0.2<br>*-0.6*	-0.9<br>*-0.7*	31.6<br>*32.0*
AG	3.5<br>*3.4*	-0.3<br>*-0.4*	-0.8<br>*-0.6*	2.4<br>*3.1*	-2.2<br>*-2.5*	33.7<br>*33.5*
AT	3.3<br>*3.3*	0.0<br>*0.0*	-0.8<br>*-0.8*	-0.9<br>*-0.5*	-0.2<br>*0.0*	31.2<br>*30.4*
CA	3.1<br>*3.1*	-0.2<br>*-0.2*	0.0<br>*-0.2*	8.6<br>*10.3*	0.5<br>*0.2*	26.5<br>*29.6*
TA	3.3<br>*3.2*	0.0<br>*0.0*	-0.4<br>*-0.2*	9.6<br>*10.0*	-0.4<br>*0.0*	32.8<br>*28.9*
CX[b]	2.9	0.7	-0.5	0.1	-2.9	19.7
XA	3.3	0.3	-0.3	0.4	2.9	35.1

XC	3.4	0.1	-0.4	3.2	-1.4	34.2
TX	3.1	0.5	-0.4	0.2	0.0	27.4

[a] Values for simulations were obtained from time averages computed for individual steps in each sequence. The values are compared with a large ensemble of MD simulations performed with parmbsc0 (italic), taken from a previous work (16). Rotational parameters are in degrees (Twist, Tilt, Roll), and translational ones (Shift, Slide, Rise) in Å. Complementary steps (e.g. AG and CT) have the same average except for a change in sign of Shift and Tilt. [b] The X base, represents all the 12 possible MMes.

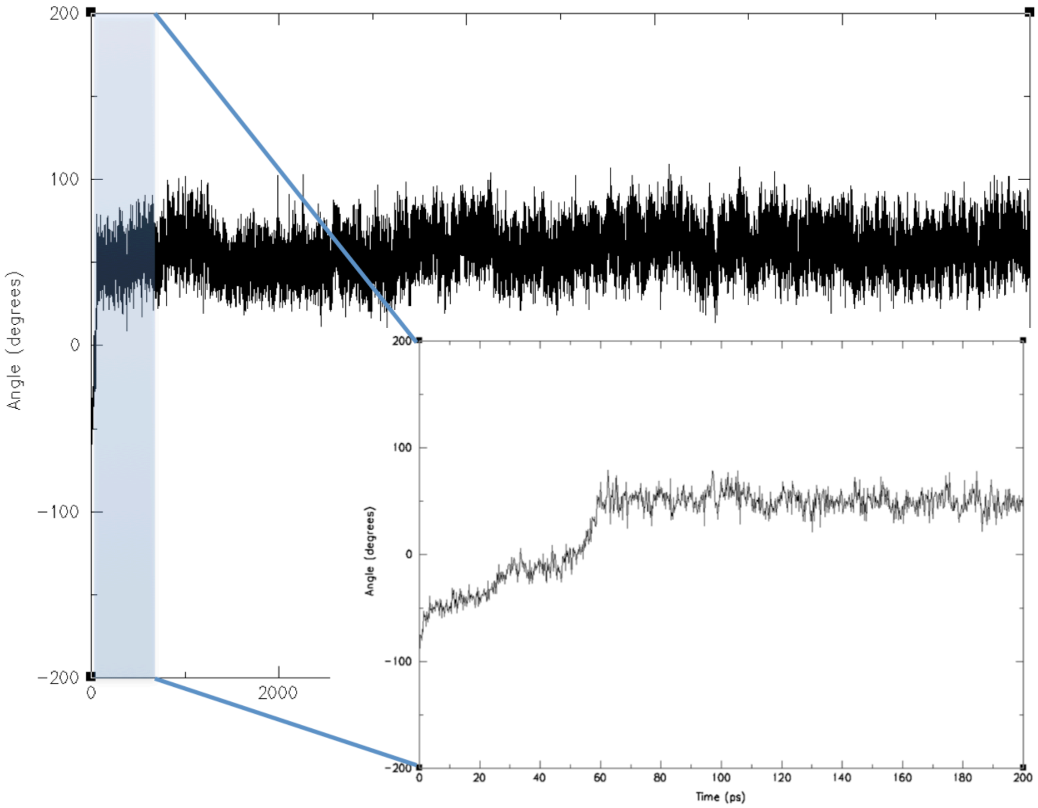

Figure S1. χ angle degree as a function of simulated time of G·G MM in the f1 environment during ABMD simulations(17).

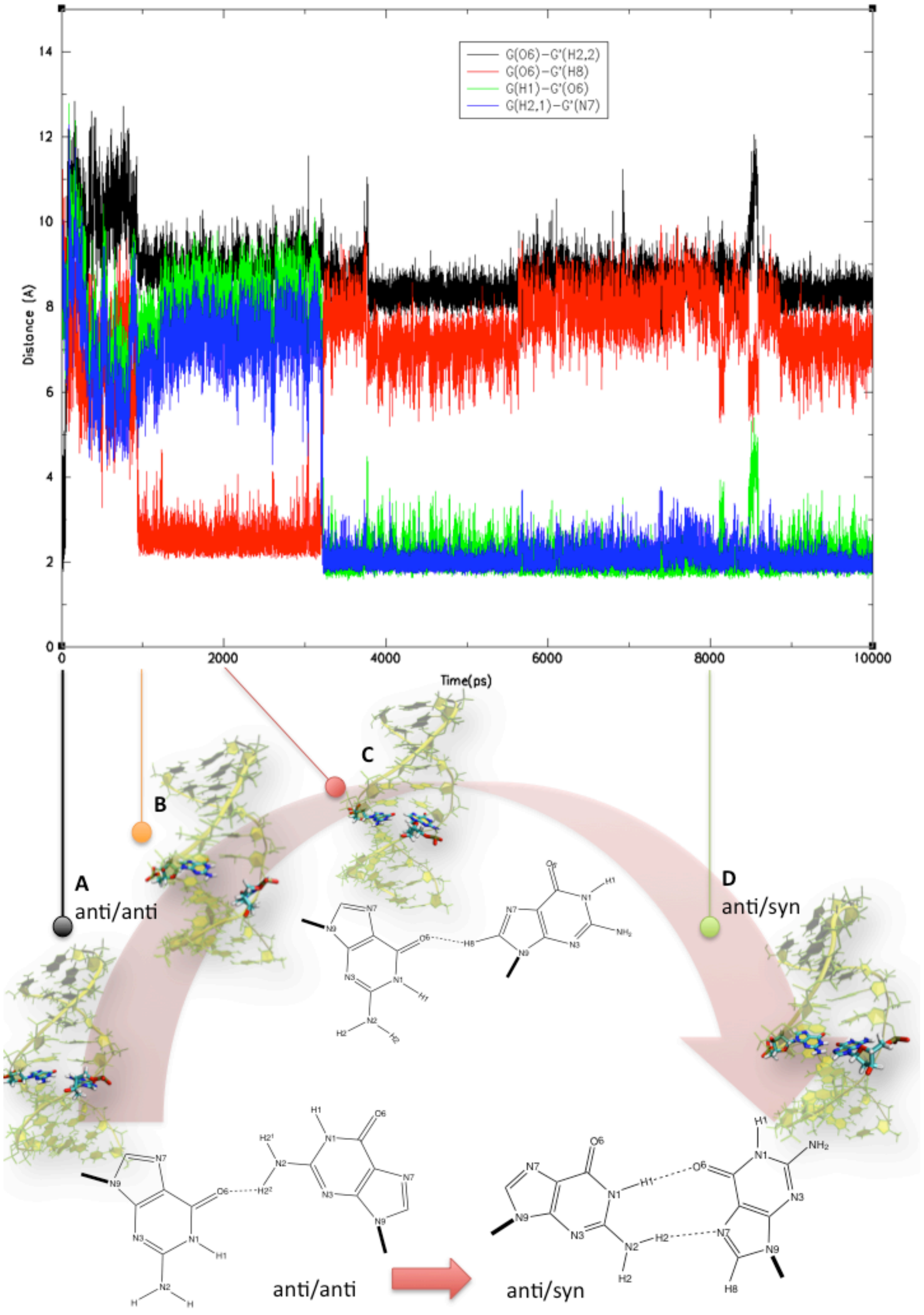

Figure S2. HB pathway as a function of simulated time. Snapshots from ABMD simulations(17) on passing from *anti* to *syn* in G·G MM in the f1 environment are shown below. The MM is highlighted in licorice, while the rest of the system is in ribbon representation**.** A schematic view of the HB network on passing from *anti* to *syn* is also reported.

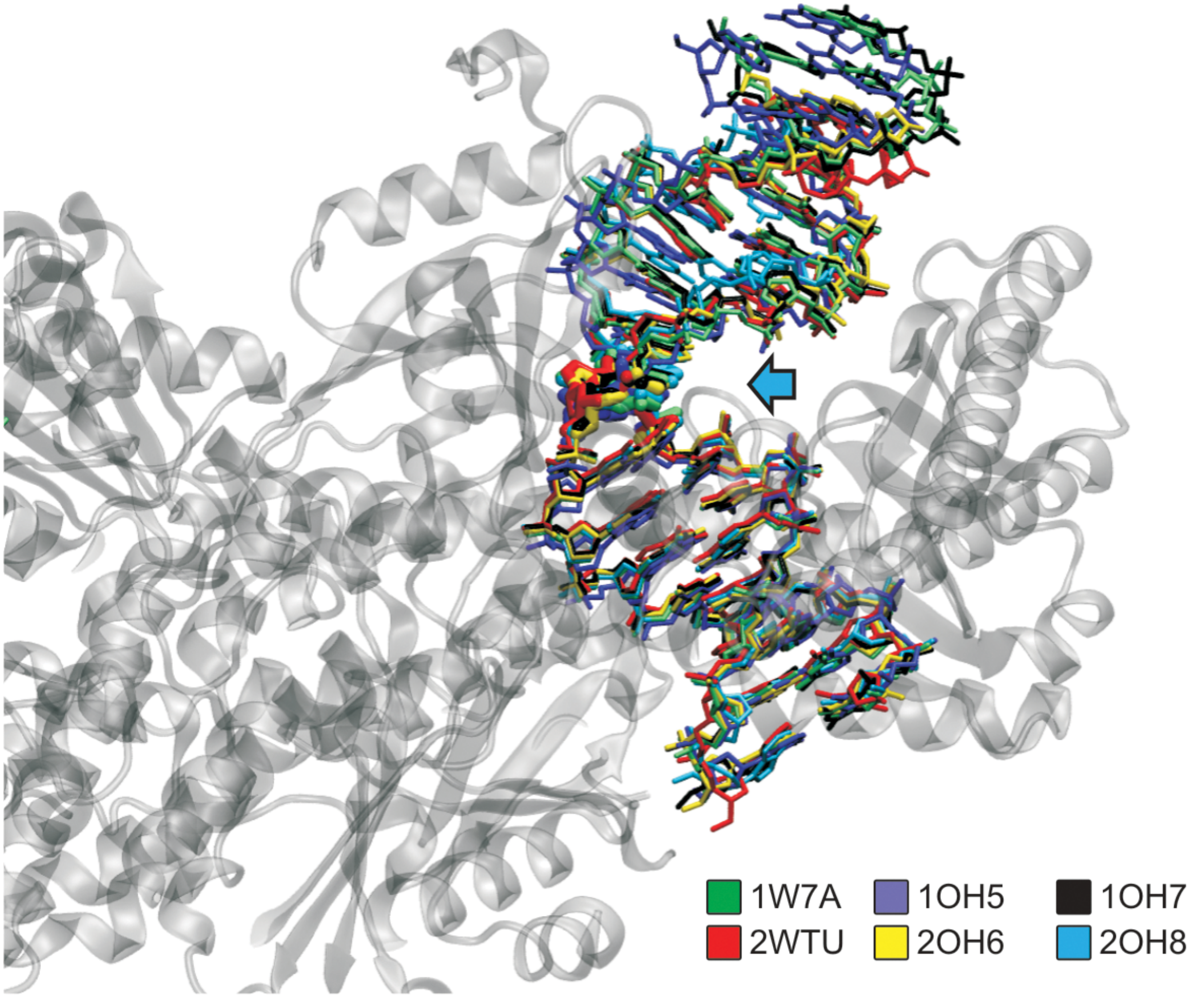

Figure S3. Structural representation of the experimentally determined MutS-DNA complexes. The six DNA segments present in the structures with PDB codes: 1W7A(18), 1OH5(19), 1OH7(19), 2WTU(20), 1OH6(19) and 1OH8(19) were aligned to highlight the global bending of the DNA. A blue arrow indicates the mismatch site, which also correspond to the hinge point of the bending observed on DNA in the MutS-DNA complexes. Note that for the sake of clarity we only show the MutS secondary structure for one of the cases (1W7A(18)).

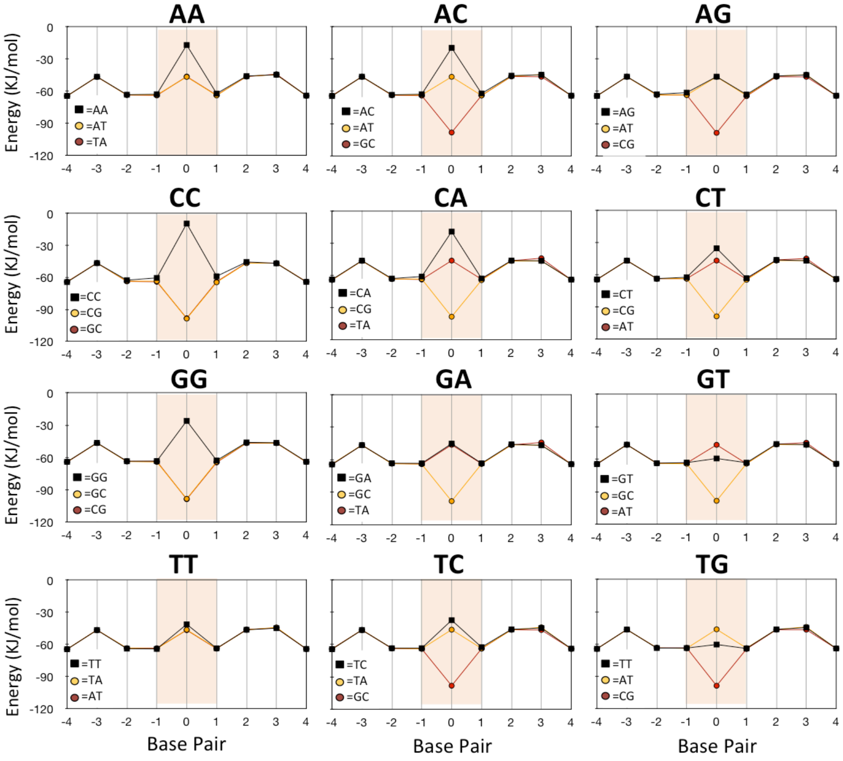

Figure S4. ***HB energy.*** The shadow region indicates the spread of the perturbation induced by the MM.

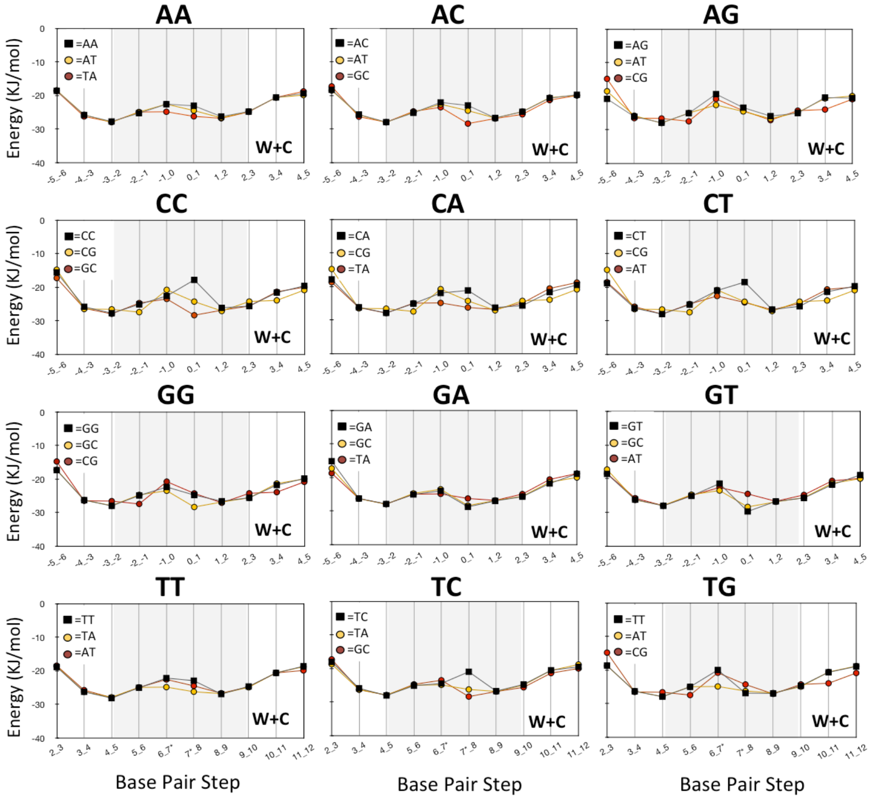

Figure S5. ***Stacking Energies*** mediated along the Watson + Crick (W+C) filaments**.** The shadow region indicates the spread of the perturbation induced by the MM.

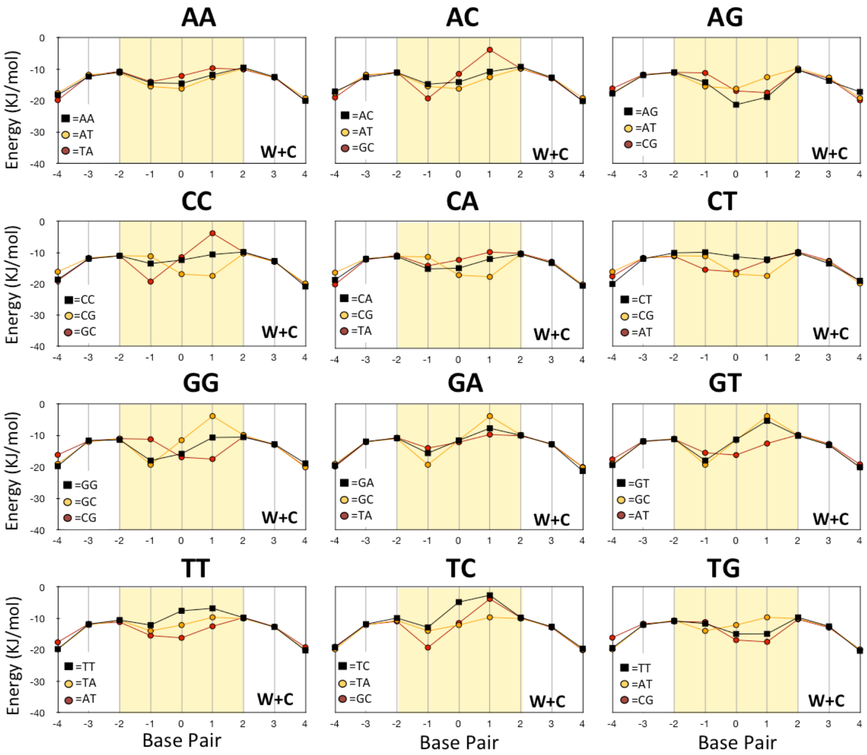

Figure S6. ***Cross Term pairing energies*** mediated along the Watson + Crick (W+C) filaments. The shadow region indicates the spread of the perturbation induced by the MM.

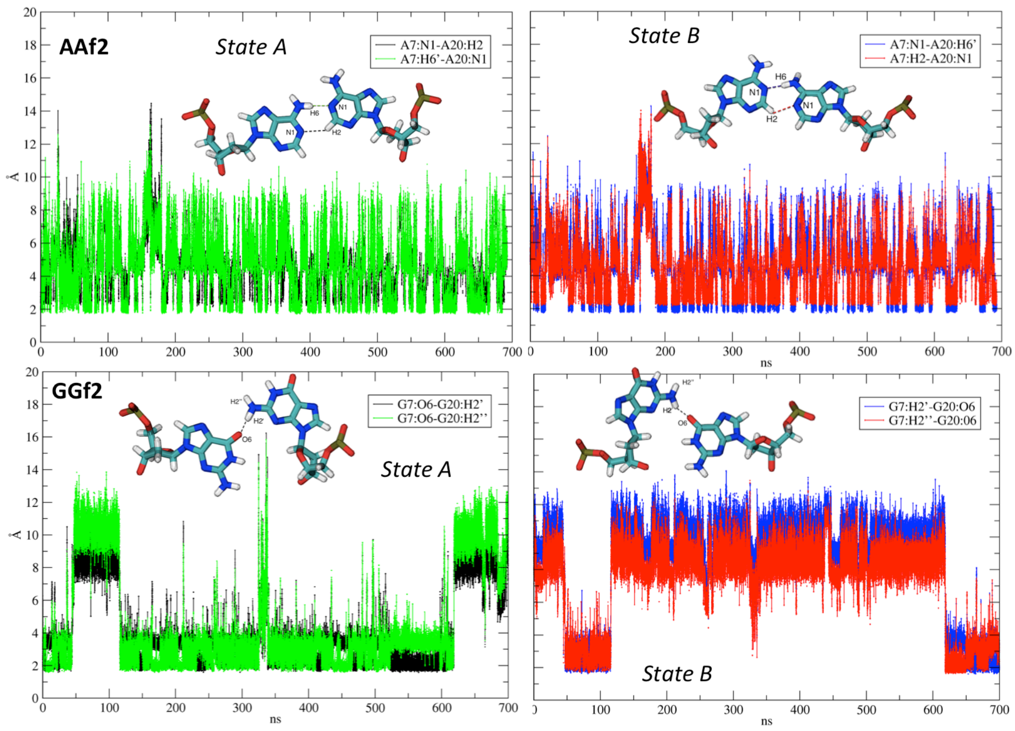

Figure S7. H-bonding scheme of AAf2 and GGf2 MMs as a function of simulated time in the extended simulations (700ns).

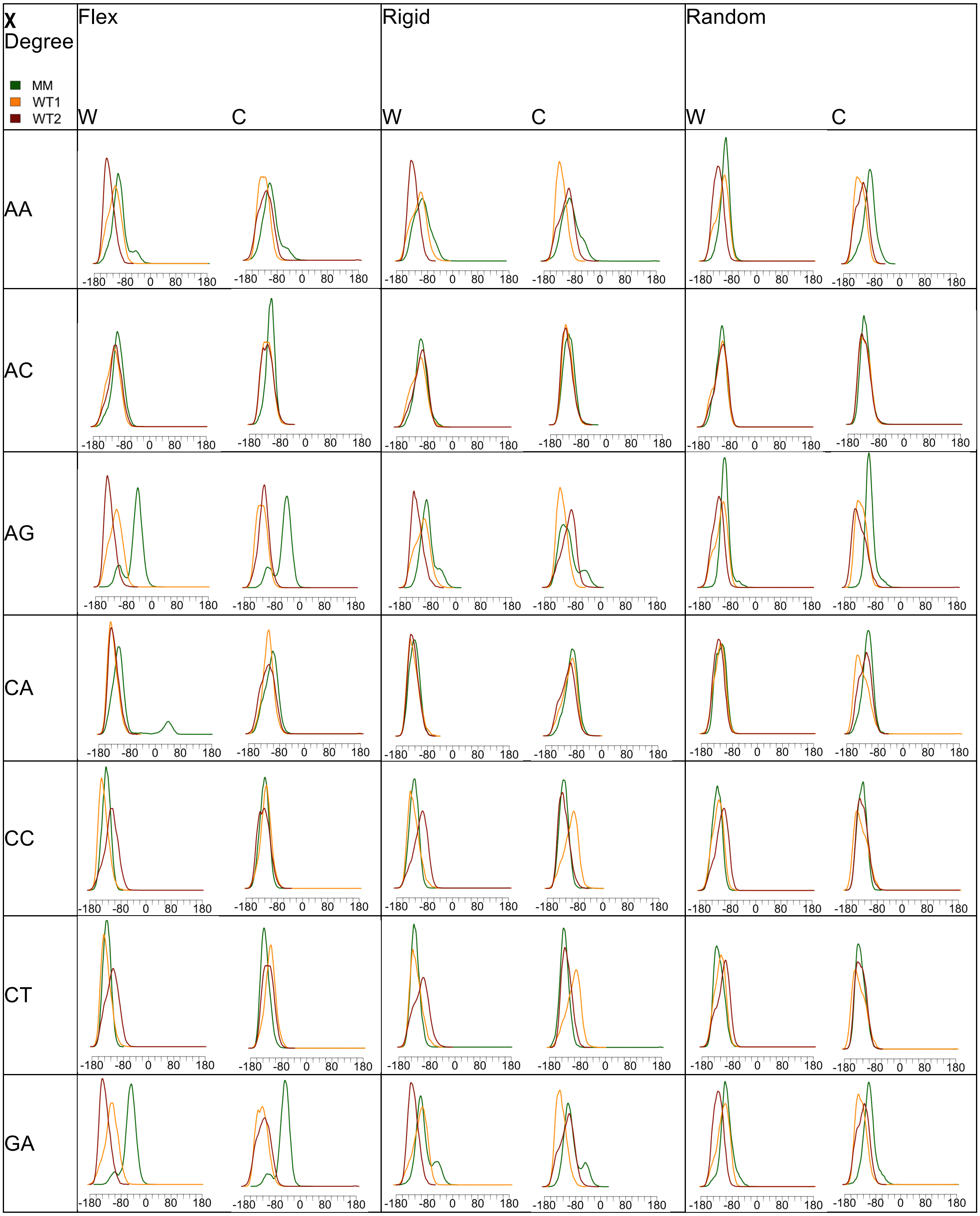
χ Degree
MM
WT1
WT2
Flex
Rigid
Random
W
C
AA
AC
AG
CA
CC
CT
GA
-180 -80 0 80 180

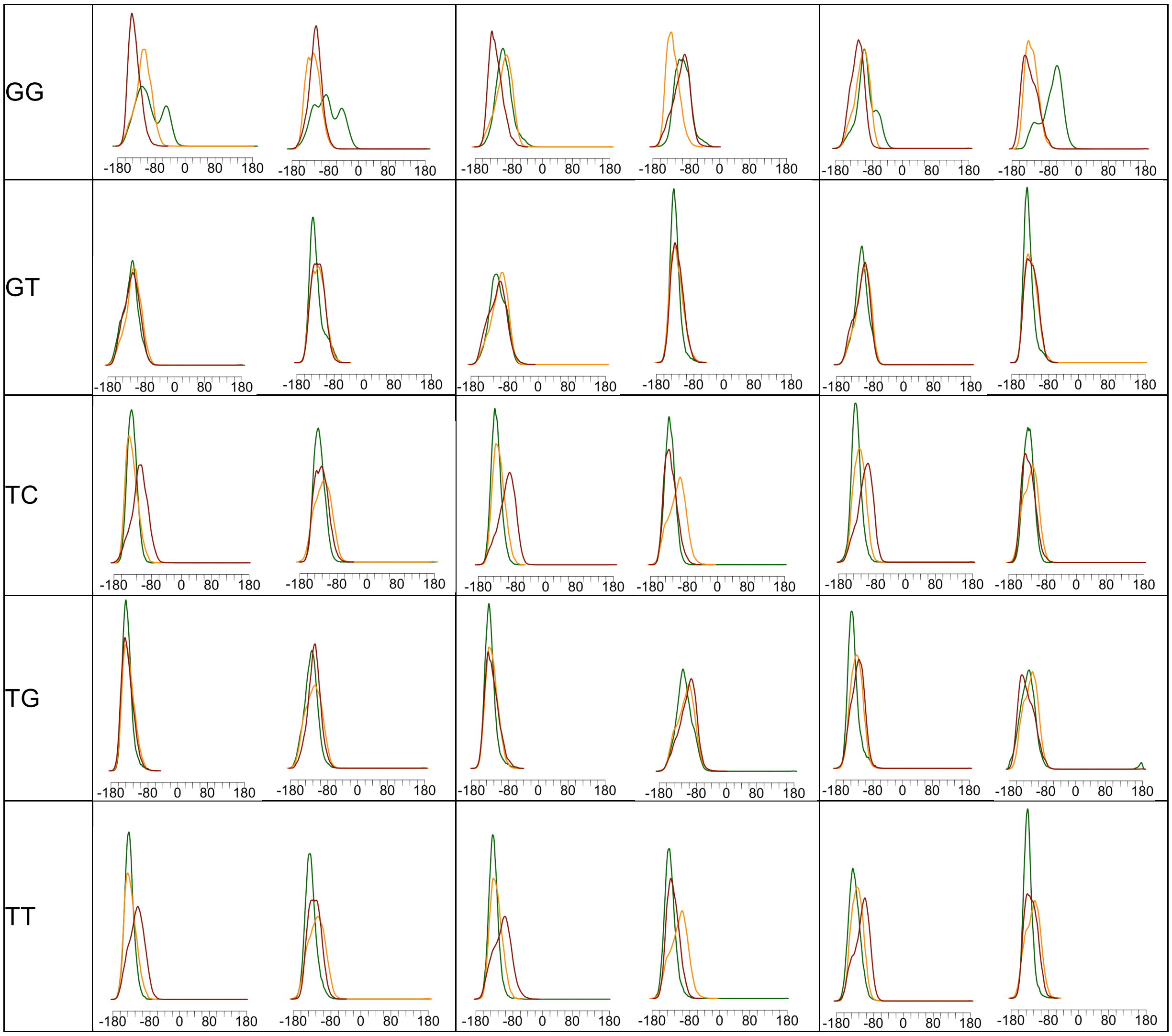

Figure S8. χ angle distributions of all the MMs during the overall simulations with respect two references (WT1, WT2). The first reference (WT1) is A:T, C·G, G·C, and T·A for A·X, C·X, G·X and T·X repspectively. The second reference (WT2) is T:A, G·C, C·G and A:T for Y·A, Y·C, Y·G and Y·T respectively. (i.e. for a given MMs, Y·X, for istance G·A, WT1=G:C, WT2=T:A).

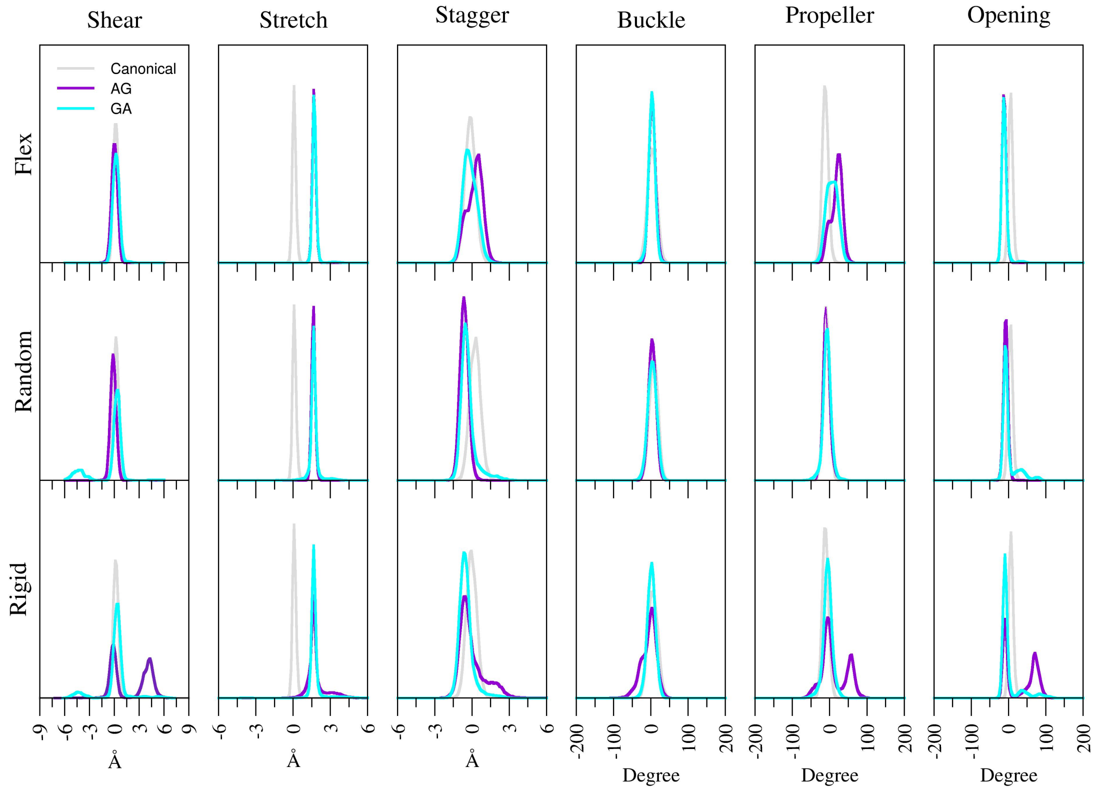

Figure S9 a: Intra base pair parameters for A·G/G·A MM

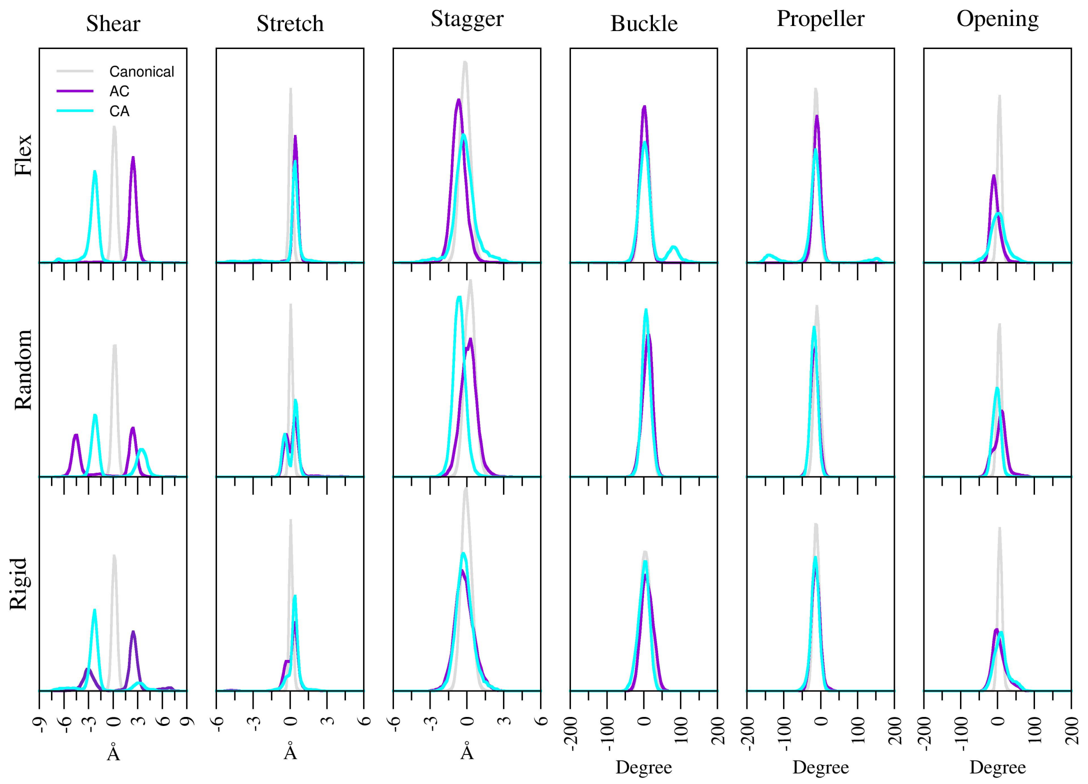

Figure S9 b: Intra base pair parameters for A·C/C·A MM

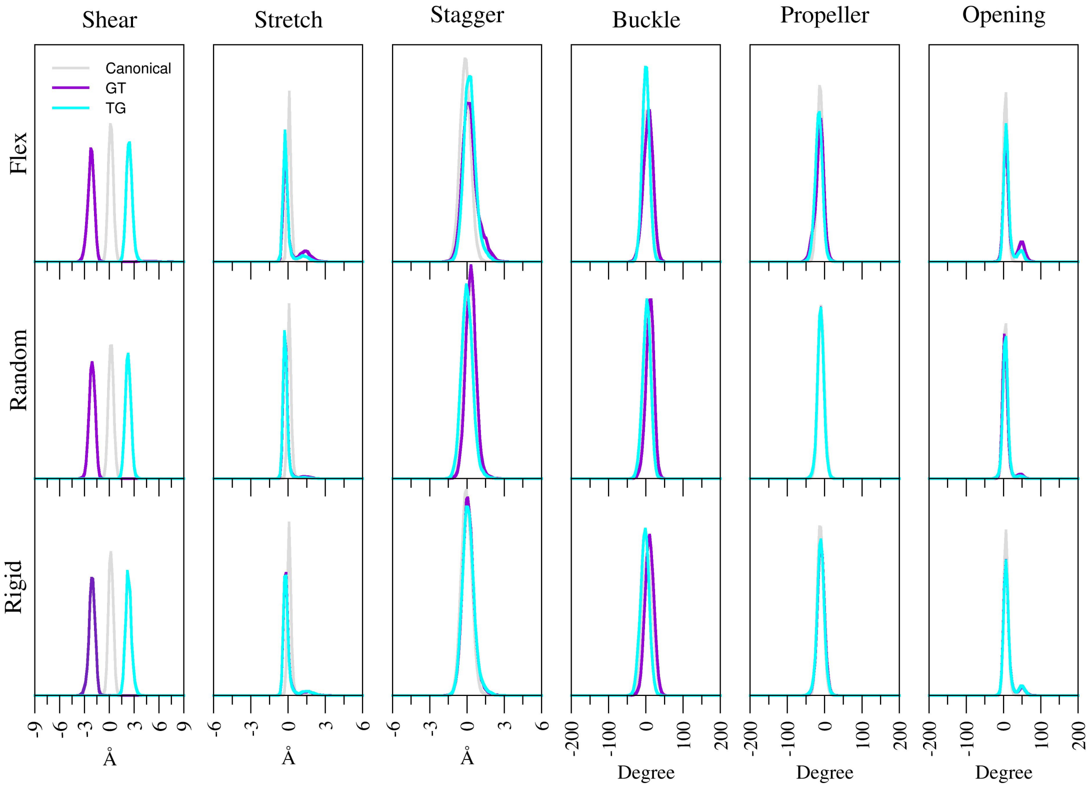

Figure S9 c: Intra base pair parameters for G·T/T·G MM

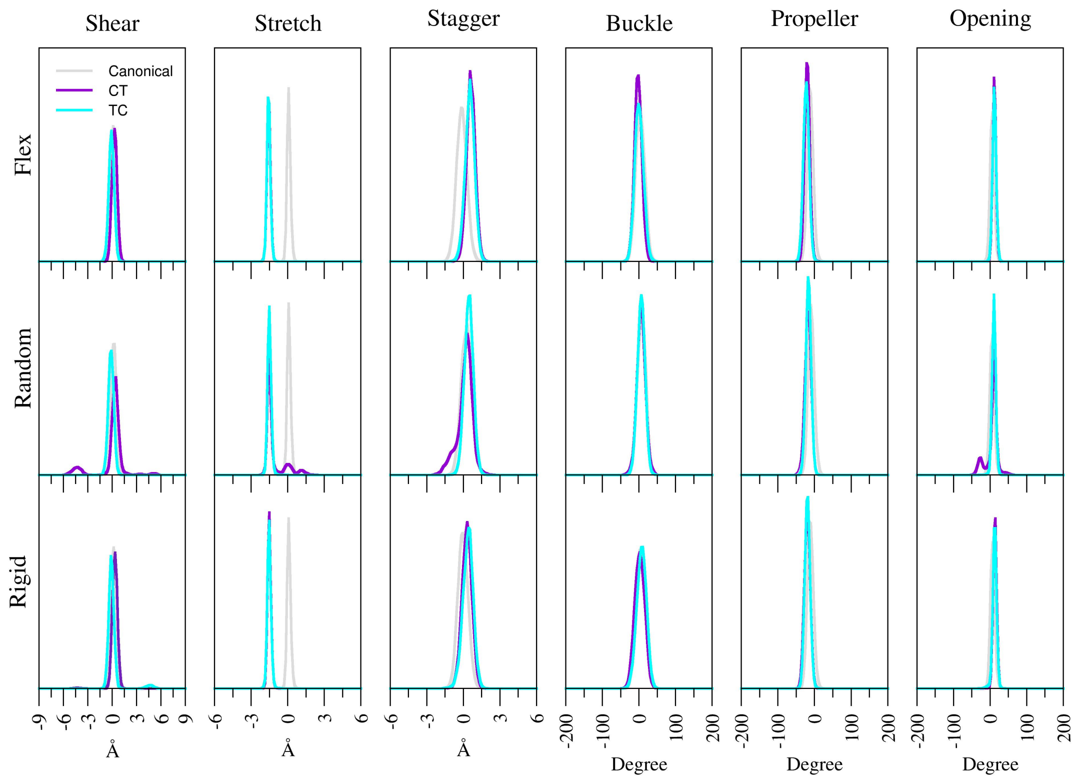

Figure S9 d: Intra base pair parameters for C·T/T·C MM

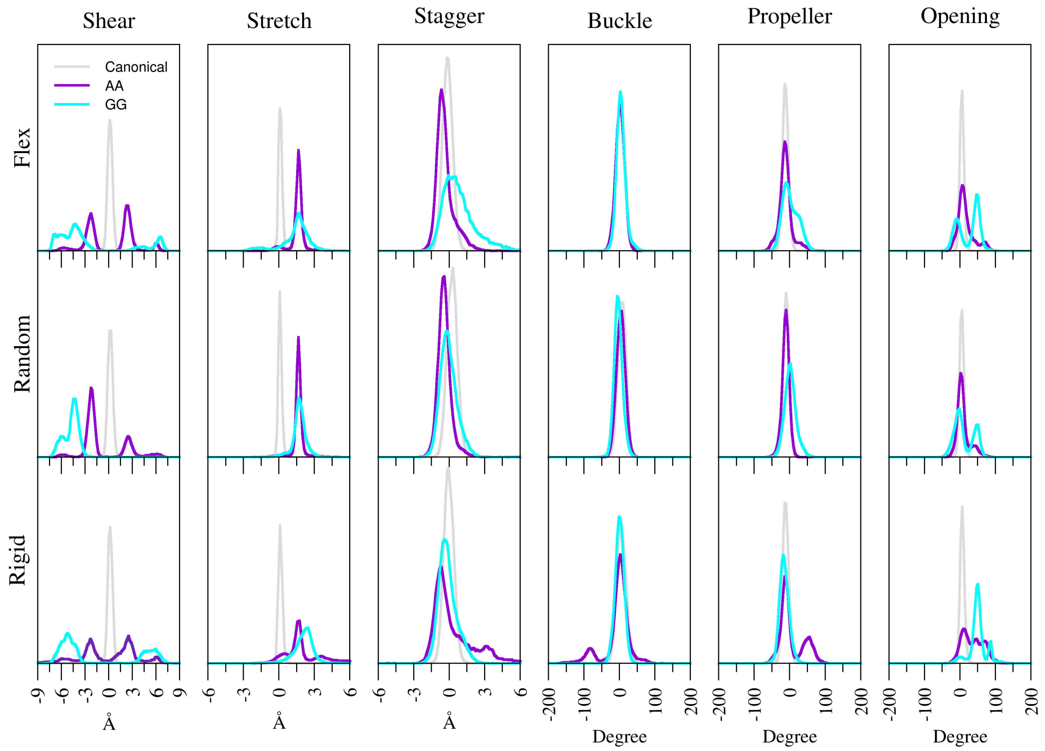

Figure S9 e: Intra base pair parameters for A·A and G·G MM

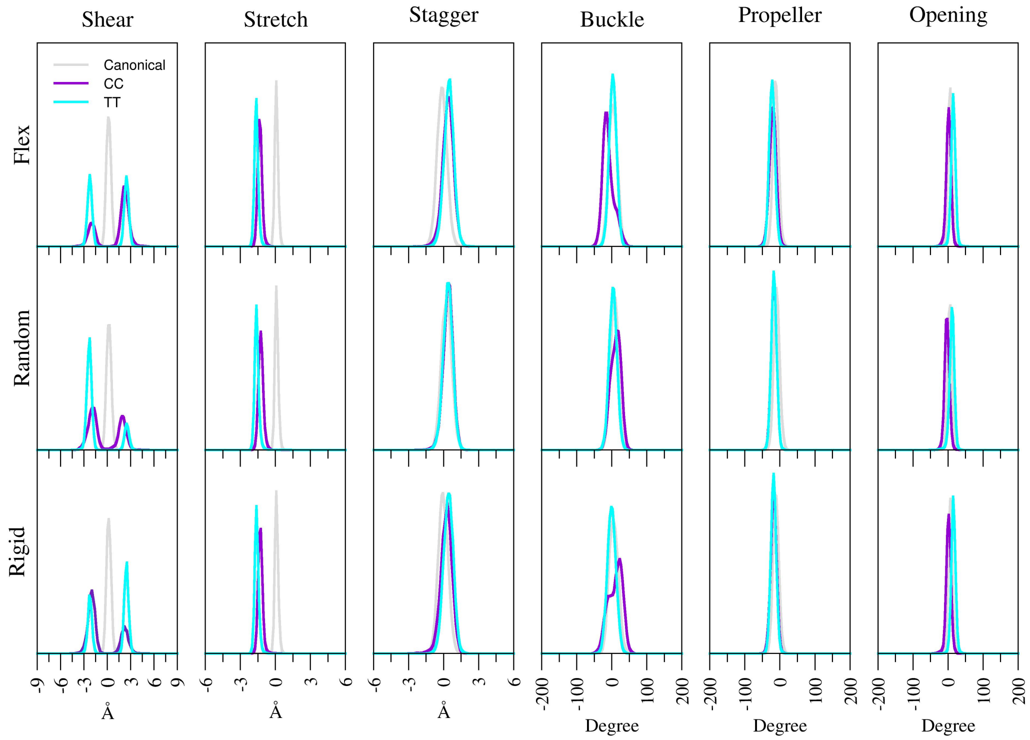

Figure S9 f: Intra base pair parameters for C·C and T·T MM

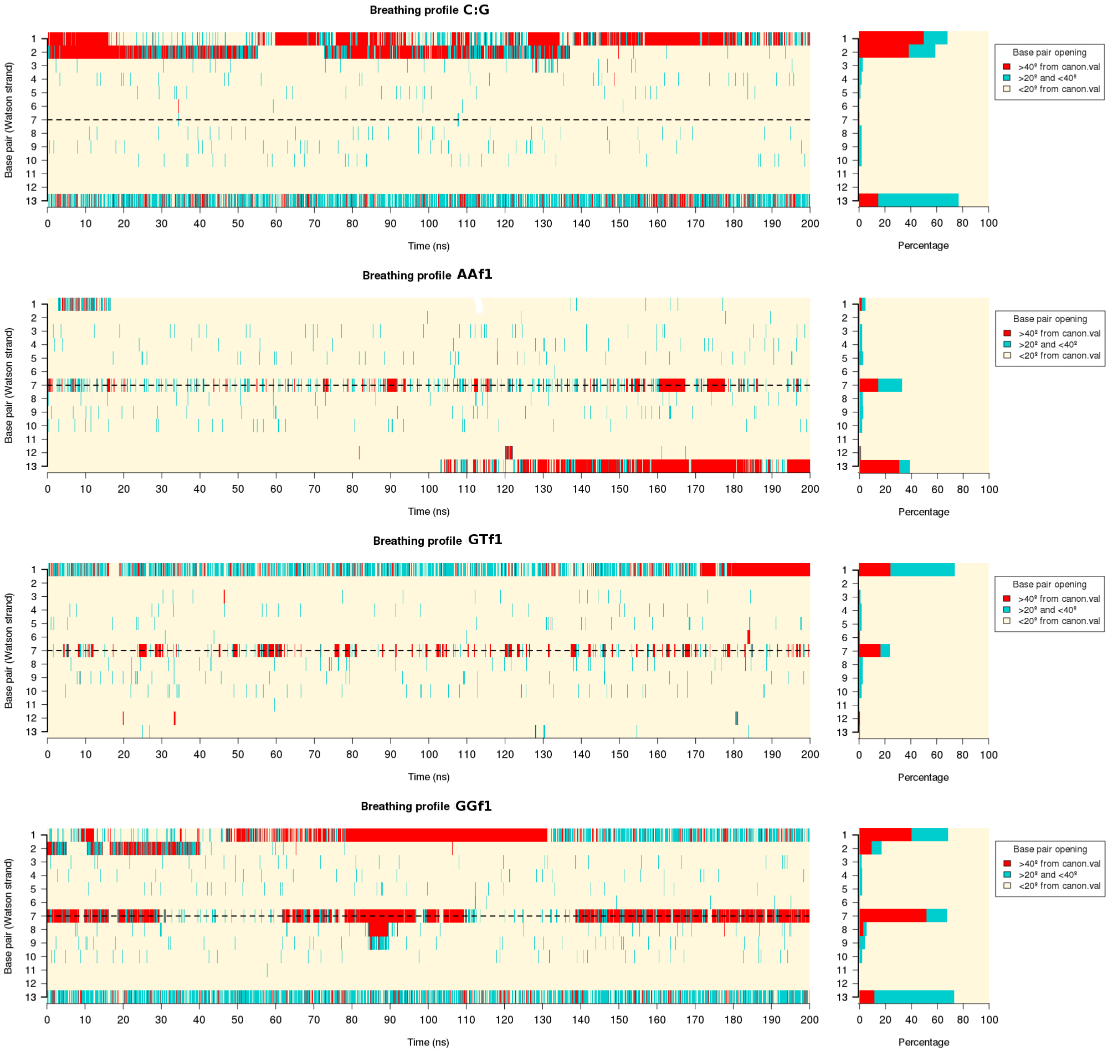

Figure S10. Breathing profile for 3 selected MMs showing low (GTf1), moderate (AAf1), and high (GGf1) breathing with respect to the canonical (C:G) pairing. The time evolution and total percentage of breathing, based on the opening helical parameter (see Methods), are reported. The position of the MM is indicated by a dashed line (base pair 7).

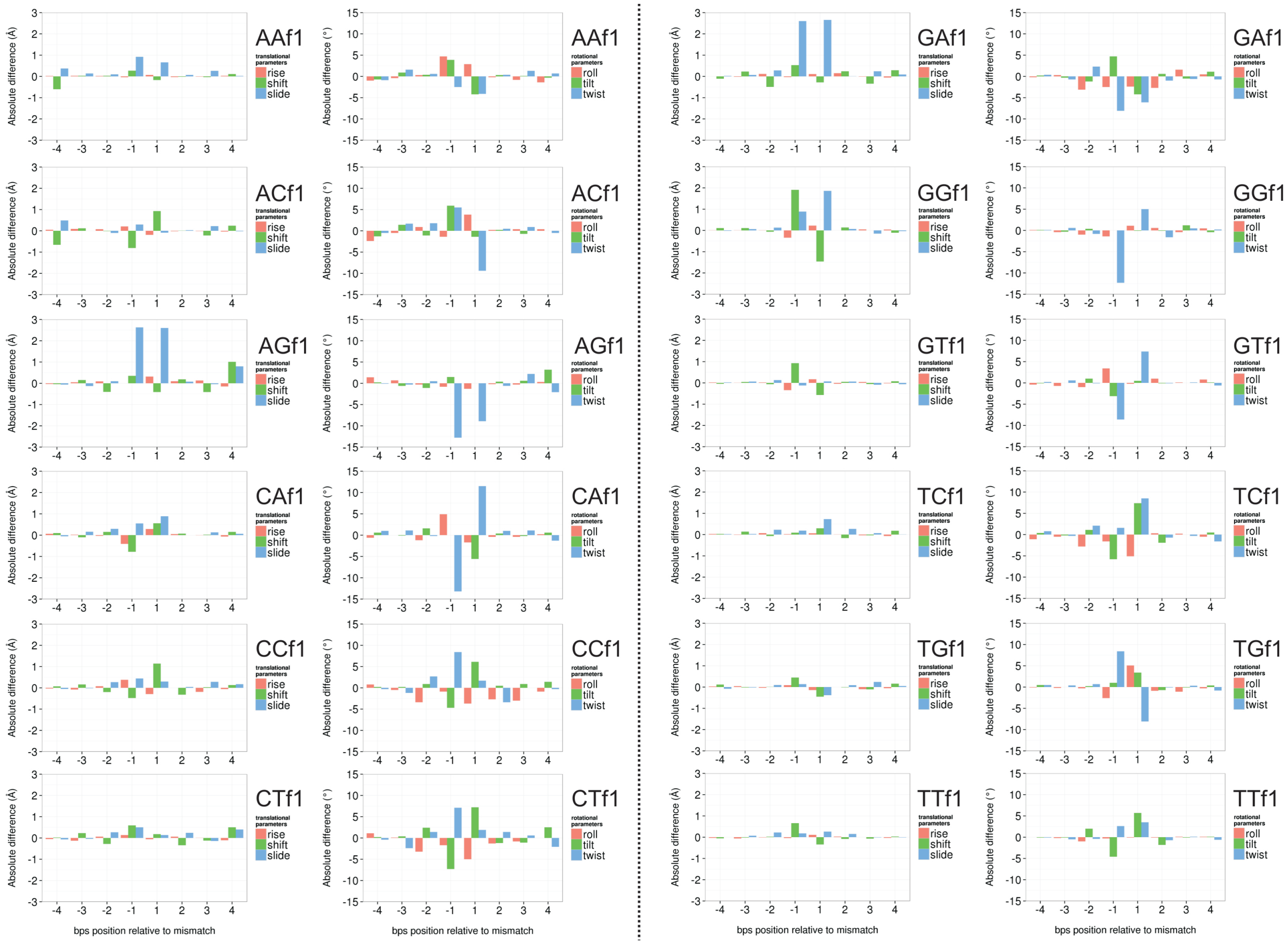

Figure S11. *Lesion information transfer*. Structural distortions induced by the MMs on the neighboring base pairs steps (bps) for all the possible MMs in the flexible environment (f1, see Figure 1). The absolute difference was obtained by subtracting the average value of a given helical parameters and a given bps in the MM simulation to the same helical parameter and bps in the canonical simulations. Note that the sequence is referred relative to the MM base pair (position 0). Translational (angstroms) and rotational (degrees) helical parameters are displayed separately.

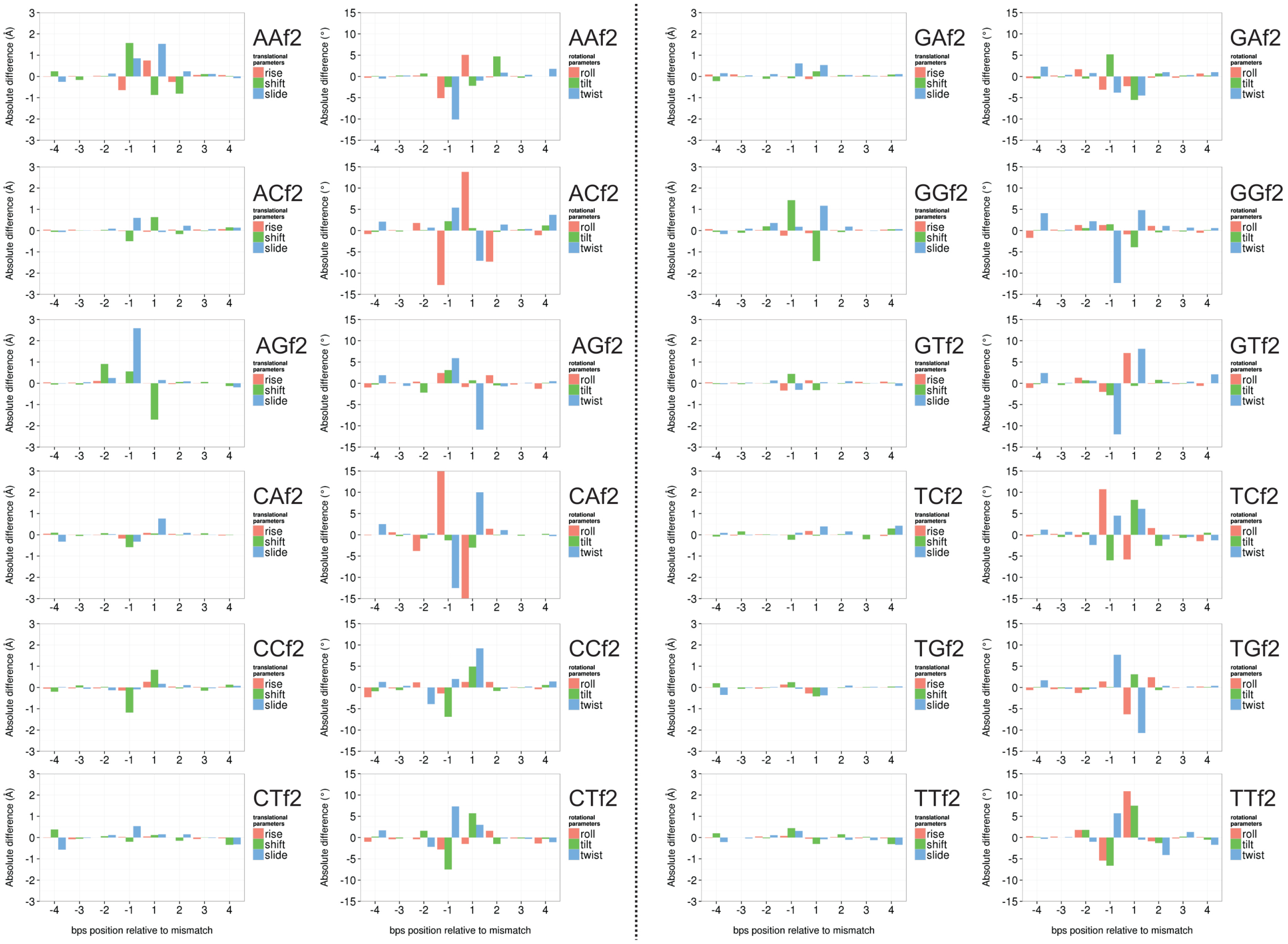

Figure S12. *Lesion information transfer*. Structural distortions induced by the MMs on the neighboring base pairs steps (bps) for all the possible MMs in the flexible environment (f2, see Figure 1). The absolute difference was obtained by subtracting the average value of a given helical parameters and a given bps in the MM simulation to the same helical parameter and bps in the canonical simulations. Note that the sequence is referred relative to the MM base pair (position 0). Translational (angstroms) and rotational (degrees) helical parameters are displayed separately.

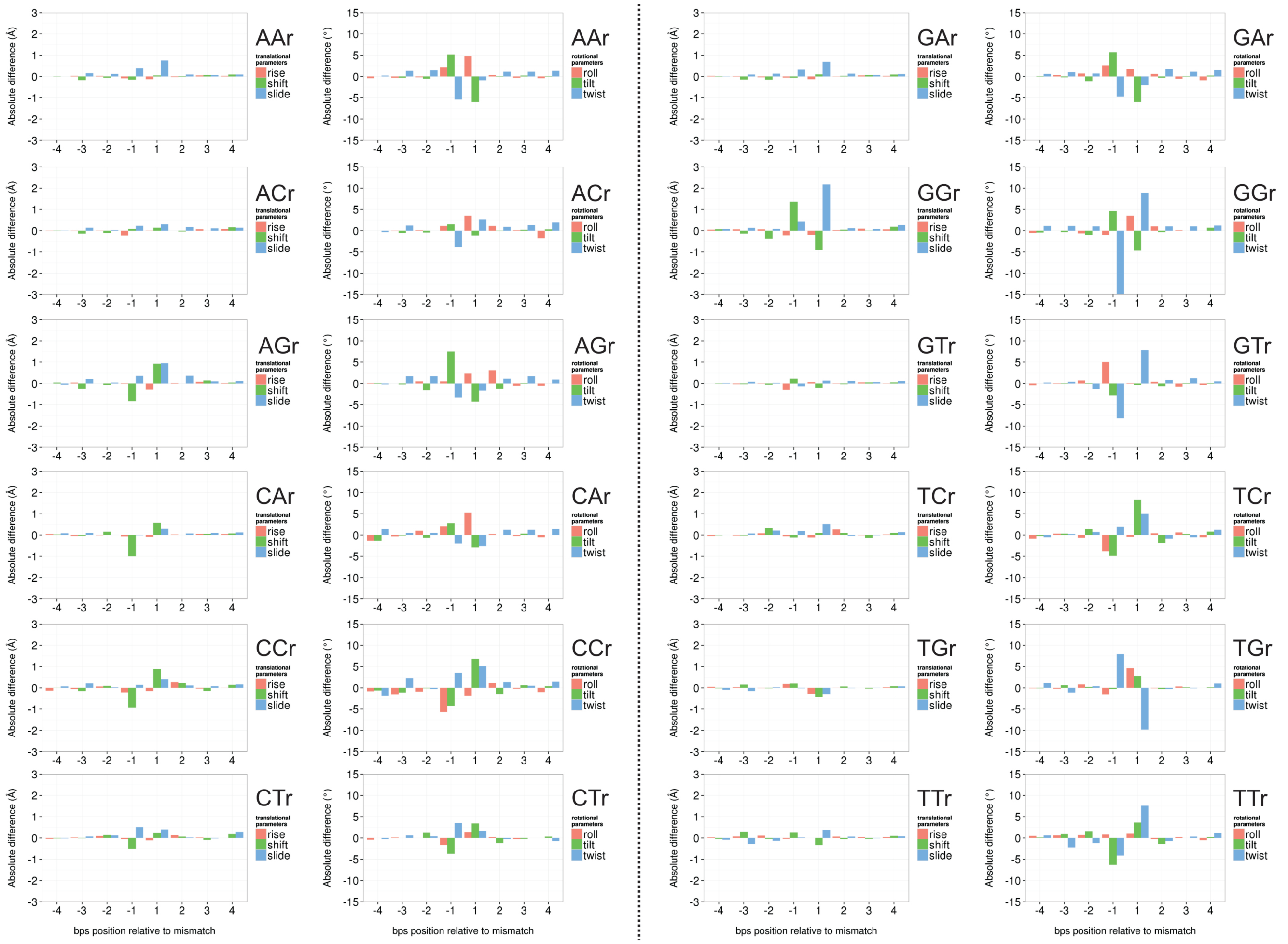

Figure S13. *Lesion information transfer*. Structural distortions induced by the MMs on the neighboring base pairs steps (bps) for all the possible MMs in the rigid environment (r, see Figure 1). The absolute difference was obtained by subtracting the average value of a given helical parameters and a given bps in the MM simulation to the same helical parameter and bps in the canonical simulations. Note that the sequence is referred relative to the MM base pair (position 0). Translational (angstroms) and rotational (degrees) helical parameters are displayed separately.

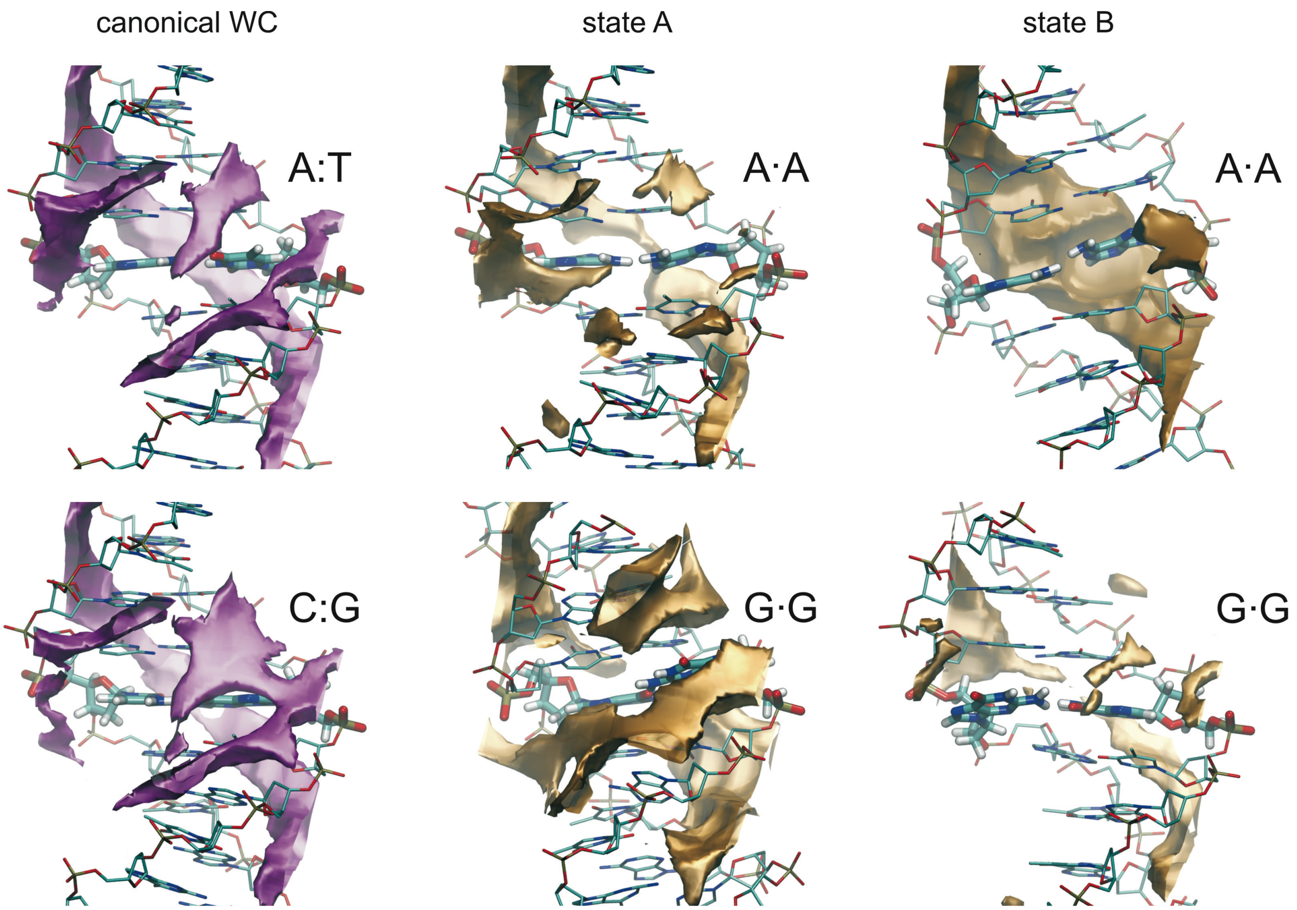

Figure S14. Altered interaction with DNA grooves at the lesion site: Classical molecular interaction potentials using sodium as a probe. For sake of comparison, the averaged structures obtained from the simulations were aligned and the same iso-surface of -7.5 kcal·mol-1 was computed. The ability of DNA to recognize sodium in the canonical Watson-Crick base pairs A:T and C:G (3D density in violet) is compared with two structures of the G·G and A·A MMs in the flexible environment (3D density in gold). The chosen structures are representative of the two HB states detected during the simulations (see Figure 5). Note that the base pair where the lesion occurs is highlighted.

REFERENCES

1. Hospital, A., Faustino, I., Collepardo-Guevara, R., González, C., Gelpí, J.L. and Orozco, M. (2013) NAFlex: a web server for the study of nucleic acid flexibility. *Nucleic Acids Res.*, **41**, W47-55.
2. Pronk, S., Páll, S., Schulz, R., Larsson, P., Bjelkmar, P., Apostolov, R., Shirts, M.R., Smith, J.C., Kasson, P.M., van der Spoel, D. *et al.* (2013) GROMACS 4.5: a high-throughput and highly parallel open source molecular simulation toolkit. *Bioinformatics*, **29**, 845-854.
3. Case, D.A., Babin, V., Berryman, J., Betz, R.M., Cai, Q., Cerutti, D.S., Cheatham, I.T.E., Darden, T.A., Duke, R.E., Gohlke, H. *et al.* (2014) *Amber 14*. University of California.

4. Blanchet, C., Pasi, M., Zakrzewska, K. and Lavery, R. (2011) CURVES+ web server for analyzing and visualizing the helical, backbone and groove parameters of nucleic acid structures. *Nucleic Acids Res.*, **39**, W68-73.
5. Lavery, R., Moakher, M., Maddocks, J.H., Petkeviciute, D. and Zakrzewska, K. (2009) Conformational analysis of nucleic acids revisited: Curves+. *Nucleic Acids Res.*, **37**, 5917-5929.
6. Lavery, R., Maddocks, J.H., Pasi, M. and Zakrzewska, K. (2014) Analyzing ion distributions around DNA. *Nucleic Acids Res.*, **42**, 8138-8149.
7. Dans, P.D., Faustino, I., Battistini, F., Zakrzewska, K., Lavery, R. and Orozco, M. (2015) Unraveling the sequence-dependent polymorphic behavior of d(CpG) steps in B-DNA. *Nucleic Acids Res.*, **42**, 11304-11320.
8. Lankas, F., Sponer, J., Hobza, P. and Langowski, J. (2000) Sequence-dependent elastic properties of DNA. *J. Mol. Biol.*, **299**, 695-709.
9. Perez, A., Lankas, F., Luque, F.J. and Orozco, M. (2008) Towards a molecular dynamics consensus view of B-DNA flexibility. *Nucleic Acids Res.*, **36**, 2379-2394.
10. Gelpi, J.L., Kalko, S.G., Barril, X., Cirera, J., de La Cruz, X., Luque, F.J. and Orozco, M. (2001) Classical molecular interaction potentials: improved setup procedure in molecular dynamics simulations of proteins. *Proteins: Struct., Funct., Bioinf.*, **45**, 428-437.
11. Orozco, M. and Luque, F.J. (2001) Theoretical methods for the description of the solvent effect in biomolecular systems. (vol 100, pg 4187, 2000). *Chem. Rev.*, **101**, 203-203.
12. Cheatham, T.E., Cieplak, P. and Kollman, P.A. (1999) A modified version of the Cornell et al. force field with improved sugar pucker phases and helical repeat. *J. Biomol. Struct. Dyn.*, **16**, 845-862.
13. Cuervo, A., Dans, P.D., Carrascosa, J.L., Orozco, M., Gomila, G. and Fumagalli, L. (2014) Direct measurement of the dielectric polarization properties of DNA. *Proc. Natl. Acad. Sci. U.S.A.*, **111**, E3624-E3630.
14. Development Core Team, R. (2005) In computing, A. I. a. e. f. s. (ed.). R Foundation for Statistical Computing, Vienna, Austria.
15. Daura, X., Gademann, K., Jaun, B., Seebach, D., Gunsteren, W. and Mark, A.E. (1999) Peptide Folding: When Simulation Meets Experiment. *Angew Chem Int Ed*, **38**, 236-240.
16. Dans, P.D., Pérez, A., Faustino, I., Lavery, R. and Orozco, M. (2012) Exploring polymorphisms in B-DNA helical conformations. *Nucleic Acids Research*, **40**, 10668-10678.
17. Marchi, M. and Ballone, P. (1999) Adiabatic bias molecular dynamics: A method to navigate the conformational space of complex molecular systems. *J. Chem. Phys.*, **110**, 3697-3702.
18. Lamers, M.H., Georgijevic, D., Lebbink, J.H., Winterwerp, H.H.K., Agianian, B., de Wind, N. and Sixma, T.K. (2004) ATP Increases the Affinity between MutS ATPase Domains: IMPLICATIONS FOR ATP HYDROLYSIS AND CONFORMATIONAL CHANGES. *J. Biol. Chem.*, **279**, 43879-43885.
19. Natrajan, G., Lamers, M.H., Enzlin, J.H., Winterwerp, H.H.K., Perrakis, A. and Sixma, T.K. (2003) Structures of Escherichia coli DNA mismatch repair enzyme MutS in complex with different mismatches: a common recognition mode for diverse substrates. *Nucleic Acids Res.*, **31**, 4814-4821.

20. Lebbink, J.H.G., Fish, A., Reumer, A., Natrajan, G., Winterwerp, H.H.K. and Sixma, T.K. (2010) Magnesium Coordination Controls the Molecular Switch Function of DNA Mismatch Repair Protein MutS. *J. Biol. Chem.*, **285**, 13131-13141.
